# Lilikoi V2.0: a deep learning–enabled, personalized pathway-based R package for diagnosis and prognosis predictions using metabolomics data

**DOI:** 10.1093/gigascience/giaa162

**Published:** 2021-01-23

**Authors:** Xinying Fang, Yu Liu, Zhijie Ren, Yuheng Du, Qianhui Huang, Lana X Garmire

**Affiliations:** Department of Biostatistics, School of Public Health, University of Michigan, 1415 Washington Heights, Ann Arbor, MI 49109, USA; Department of Computational Medicine and Bioinformatics, University of Michigan, 1600 Huron Parkway, Ann Arbor, MI 48105, USA; Department of Electric Engineering and Computer Science, 2260 Hayward Street, University of Michigan, Ann Arbor, MI 48109, USA; Department of Biostatistics, School of Public Health, University of Michigan, 1415 Washington Heights, Ann Arbor, MI 49109, USA; Department of Biostatistics, School of Public Health, University of Michigan, 1415 Washington Heights, Ann Arbor, MI 49109, USA; Department of Computational Medicine and Bioinformatics, University of Michigan, 1600 Huron Parkway, Ann Arbor, MI 48105, USA

**Keywords:** classification, prognosis, survival analysis, neural network, deep learning, metabolomics, pathway, visualization

## Abstract

**Background:**

previously we developed Lilikoi, a personalized pathway-based method to classify diseases using metabolomics data. Given the new trends of computation in the metabolomics field, it is important to update Lilikoi software.

**Results:**

here we report the next version of Lilikoi as a significant upgrade. The new Lilikoi v2.0 R package has implemented a deep learning method for classification, in addition to popular machine learning methods. It also has several new modules, including the most significant addition of prognosis prediction, implemented by Cox-proportional hazards model and the deep learning–based Cox-nnet model. Additionally, Lilikoi v2.0 supports data preprocessing, exploratory analysis, pathway visualization, and metabolite pathway regression.

**Conculsion:**

Lilikoi v2.0 is a modern, comprehensive package to enable metabolomics analysis in R programming environment.

## Introduction

Metabolomics is an increasingly popular platform to systematically investigate metabolites as potential biomarkers for diseases [1]. With the rapid development in this field, data analysis is becoming a critical component to interpret and apply the results for translational and clinical research. However, currently the majority of metabolomics analysis workflows are provided as web applications [1], limiting its adaptation by the bioinformatics community, and/or integration with other omics workflows in a programmable manner.

To address such needs, previously we developed Lilikoi, a personalized pathway-based method to classify diseases using metabolomics data [2]. Different from other metabolomics analysis packages, the personalized and pathway-based representation of metabolomics features is the highlight of Lilikoi version 1 (v1). Lilikoi v1 enables classifications using various machine learning methods. It has 4 modules: feature mapper, dimension transformer, feature selector, and classification predictor [2].

Here we report Lilikoi v2.0, a significant upgrade for Lilikoi v1. The update was sparked by several recent trends or needs in the research community. First, given the recent applications of deep learning in the metabolomics and other genomics fields [3–9], it is important to enable metabolomics researchers to investigate such new approaches. We thus implemented a deep learning neural network as a new method in the classification module. Second, metabolomics data have the potential to be prognosis markers [10]; however, at present it is rare for a metabolomics data analysis workflow to be available for prognosis modeling and prediction. We herein implemented multiple methods for prognosis prediction, including a Cox–proportional hazards (Cox-PH) model and Cox-nnet, a neural network–based model [5]. Third, we augmented the pathway-based metabolomics analysis with metabolite-pathway regression and pathway visualization. Last, we also include additional preprocessing methods for metabolomics data analysis (e.g., normalization, imputation) and tools for exploratory data analysis (e.g., principal component analysis [PCA], t-distributed stochastic neighbor embedding [t-SNE] analysis, and source of variation [SOV] analysis). In summary, Lilikoi v2.0 is a more mature, comprehensive, and modern package to empower the metabolomics community.

## Methods

### Datasets

Three breast cancer metabolomics datasets were used to demonstrate the new functionalities of Lilikoi v2.0. The first set was downloaded from the Metabolomics Workbench project ID PR000284 [11], which used 207 plasma samples (126 breast cancer cases and 81 control cases) with 227 metabolites from a previous study [2]. Gas chromatography–mass spectrometry and liquid chromatography–mass spectrometry metabolomics profiling were used to generate the dataset. The second metabolomics dataset is from a biobank at the Pathology Department of Charité Hospital, Berlin, Germany. It contains 162 metabolites from 271 breast cancer samples, where 204 samples are estrogen receptor positive (ER+) and 67 samples are ER− [12]. Metabolomics in this dataset were based on gas chromatography–time-of-flight mass spectrometry. The third dataset was shared by authors from an original National Cancer Institute (NCI) study, composed of 536 metabolites from 67 breast tumor samples and 65 tumor-adjacent noncancerous tissues [10]. In our analysis, we only used the 67 breast tumor samples for prognosis modeling.

### Data preprocessing

For data preprocessing, we added normalization and imputation methods. Three normalization methods (standard normalization, quantile normalization, and median-fold normalization) were implemented. We used the normalize.quantiles function in the preprocessCore package [13] to perform the quantile normalization. For imputation, we the used *k*-nearest neighbors (knn) method as the default method to impute missing values. The knn imputation was performed by the impute.knn function in the impute R package [14].

### Exploratory analysis

PCA is a feature selection technique [15] that extracts the most important information in high-dimensional datasets. The t-SNE plot is a dimension reduction method that helps users visualize high-dimensional data [16]. We implemented the PCA and t-SNE plots in Lilikoi v2.0 via the M3C package [17]. We also added SOV for exploratory analysis, implemented by the Anova function in the car package [18]. SOV identifies the relationships between confounders and metabolomics data, based on ANOVA tests [19,20]. Any clinical variable with F-score bigger than the error term, whose F-score is 1, is deemed a confounder.

### Metabolite- to pathway-level transformation

Most other pathway analysis tools for metabolomics data use the Fisher exact test or hypergeometric test, and their performance has been compared previously [21]. Different from all these methods, Lilikoi uses the Pathifier algorithm to perform the metabolites-pathway dimension transformation per sample [22]. For each pathway *P* in each patient *i*, a pathway dysregulation score (PDS) *D_P_*(*i*) with a value between 0 and 1 is generated on the basis of the metabolites associated with this pathway. A larger PDS value represents a higher degree of dysregulation (larger deviation from the normal controls). As the result of the dimension transformation, a new pathway-level matrix is constructed, which can be used to substitute the original metabolomics profile matrix, for downstream classification or prognosis modeling.

Briefly, the PDS score *D_P_*(*i*) is calculated as follows: in the high-dimensional space *d_P_* composed of metabolite vectors (where each metabolite belongs to pathway *P*), all samples form a data cloud, where sample *i* is a data point *x_i_*. The principal curve *S_P′_* in this space *d_P_* is then computed using the algorithm of Hastie and Stuetzle [23]. For each sample, the data point *x_i_* is projected onto the principal curve *S_P′_*. The dysregulation score *D_P_*(*i*) of sample *i* is then defined as the distance from the start of the principal curve to the projected point on this curve. More details of applications of Pathifier on biomarker studies (prognosis or diagnosis) can be found in earlier publications [2,24,25].

### Deep learning for classification

The deep learning algorithm in Lilikoi v2.0 is based on the H2O package [26]. It uses a multi-layer neural network trained with stochastic gradient descent search to predict the diagnosis results. For the neural network configuration, users are free to set parameters including activation function, hidden layer size, dropout ratio, L1 and L2 regularization, batch size, and adaptive learning rate decay factor. Users can also incorporate other control parameters like random discrete to optimize the hyperparameter setting to achieve the best deep learning performance.

Lilikoi v2.0 supports users to run hyperparameter grid search on multiple deep learning models to achieve the best classification results. The activation functions are set as “Rectifier” or “Tanh.” Seven hidden-layer configurations are preset for selections: 1 hidden layer setting (100 or 200 neurons), 2 hidden layer setting (10, 20, or 50 neurons for each layer), 3 hidden layers with 30 neurons for each, and 4 hidden layers with 25 neurons for each. The input dropout ratio options range from 0 to 0.9 with 0.1 increment. The number of global training samples per iteration is set to 0 or −2, where 0 means 1 epoch and −2 means the automatic value selected by the H2O package. The maximum number of times to iterate the whole dataset (epochs) is set as 500. The starting value of momentum is 0 or 0.5 (default 0, without hyperparameter grid search). The momentum damps the oscillation to achieve the optimal point and accelerates the iterations for faster convergence. The adaptive learning rate decay factor (ρ) is 0.5 or 0.99 (default 0.99, without hyperparameter grid search). The quantile value (quantile_alpha value in H2O), when running quantile regression, is set between 0 and 1. Quantile regression is similar to linear regression but measures the conditional quantile rather than the conditional mean of the response variable. The threshold between quadratic and linear loss (huber_alpha value in H2O) is set between 0 and 1 (default 0.9). The “RandomDiscrete” strategy is used to enable search on all combinations of the hyperparameters. As part of the automatic machine learning training, the maximum number of models for each run is set to 100. The training steps stop if the misclassification values do not improve by 0.01 after 5 iterations. Score_duty_cycle, the frequency of computing validation metrics, is set to 0.025 in Lilikoi v2.0, meaning that no more than 2.5% of the total training time should be used to build the validation metrics.

For the exemplary ER dataset, after grid search, the final hyperparameters for its deep learning model are set as the following: “Rectifier” activation function, 4 hidden layers with 25 neurons each, input dropout ratio 0, default training samples per iteration per H2O (value of −2), epoch value of 430.9, momentum starting value 0, ρ value of 0.99, quantile regression value of 1, and a Huber α-value of 0, other hyperparameters including an L1 regularization value of 2.5e−5, and an L2 regularization value of 2.6e−5.

This deep learning algorithm is added in classification along with 6 other machine learning techniques previously implemented in Lilikoi v1, namely, generalized boosted model (GBM), linear discriminant analysis (LDA), logistic regression (LOG), random forest (RF), recursive partitioning and regression analysis (RPART), and support vector machine (SVM). On the basis of the data and sample size, users are free to choose which algorithms they would like to use. An n-fold cross-validation (default n = 10) is applied to avoid overfitting. Classification metrics such as accuracy, F1 statistic, balanced accuracy, sensitivity (SEN), and specificity (SPEC) are reported as bar plots.

The running time for each classification method is calculated with the Sys.time() function in R and measured using the slurm job scheduler on a dedicated group computer server cluster (consisting of 4 nodes [Dell PowerEdge C6420] of 2 X Intel® Xeon® Gold 6154 CPUs at 3.00 GHz, 192 GB RAM). One processor and 50 GB memory were reserved for each job.

### Prognosis prediction

Lilikoi v2.0 enables prognosis prediction, at either the metabolite level using metabolite-sample matrix or the pathway level (after pathifier-based pathway transformation) using PDS-sample matrix. PDS is a normalized score in the range [0,1] that measures the degree of dysregulation of a pathway relative to the norm (controls). Currently 2 prognosis prediction methods are implemented: Cox-PH method [27] with penalization and the neural network–based Cox-nnet method [5]. Cox-PH is a survival regression model developed by David Cox in 1972. The input parameters are event (e.g., death), survival time, and penalized covariates: α to determine which penalization method to use and λ (lambda.min or lambda.1se) for prediction. Penalization is achieved by Lasso, Ridge, or Elastic net with the glmnet package [28]. The default λ-parameter for prediction in Cox-PH is lambda.1se. The default penalization method α is 1, which is the Lasso penalization.

Cox-nnet is based on the artificial neural network framework with a default of 2-layer neural network: a hidden layer and an output layer [29]. The output layer is fit to the Cox regression. Lilikoi v2.0 imports Cox-nnet originally written in Python, using the reticulate package.

The hazard function of the Cox-PH model is:

$h(t|{x_i}) = {h_0}( t )\mathrm{exp}( {{\theta _i}} )$ with the log hazard ratio of ${\theta _i} = {x_i}^T\beta $with its partial likelihood cost function:

\begin{equation*} \mathrm{pl}\left( \beta \right) = {\Sigma _{C\left( i \right) = 1}}\left[ {{\theta _i} - \mathrm{log}{\Sigma _{{t_i} \ge {t_j}}}\mathrm{exp}\left( {{\theta _j}} \right)} \right]. \end{equation*}

The Cox-nnet expands the Cox-PH function above as

\begin{equation*} {\theta _i} = G{(W{x_i} + b)^T}\beta , \end{equation*}

where ${x_i}$ is the output of the hidden layer, *G* is the activation function, and *W* is the coefficient weight matrix between the input and hidden layer.

\begin{equation*} \mathrm{Cost}\left( {\beta ,W} \right) = \mathrm{pl}\left( {\beta ,W} \right) + \lambda \left( {\left| {\left| \beta \right|{|_2} + } \right|\left| W \right|{|_2}} \right). \end{equation*}

In the demonstration NCI data, we applied cross-validation on the training dataset to determine the optimal L2 regularization λ-parameter. Cox-nnet supports 3 gradient descent algorithms: standard gradient descent, Nesterov accelerated gradient descent, and momentum gradient descent. The default algorithm for Cox-nnet is standard gradient descent. The hyperparameters can be set by users, including the gradient descent algorithm, initial learning rate, proportion of momentum, decrease of the learning rate, increase of the learning rate, number of iterations between cost functions to determine increase or decrease of the learning rate, maximum number of iterations, stopping threshold, minimum number of iterations before stopping, number of iterations for new lowest cost before stopping, and the random seed. Details can be found in the user manual.

The prognosis model is visualized by Kaplan-Meier curve plot, using the survminer package [30]. Samples are dichotomized into different risk groups by prognosis index (PI), the logarithm of the hazard ratio of the prognosis model. Lilikoi v2.0 allows several approaches for dichotomization: median PI threshold, event/non-event ratio, and quartile PI threshold (samples with PIs under the first quartile as the low-risk group and those above the third quartile as the high-risk group). The default dichotomous method in Lilikoi v2.0 is median PI threshold.

The fitness of the models is evaluated by 2 metrics: C-index and log-rank *P*-values. C-index is a goodness-of-fit measure of survival models [31]. A C-index of 1 indicates that the model is the best model for prediction and C-index = 0.5 means that the model prediction is no better than a random guess. Log-rank *P*-value is based on the log-rank test [32,33] to evaluate the null hypothesis that no difference in survival exists between the high-risk and low-risk groups. Log-rank *P*-value <0.05 means that there is significant difference between these 2 groups. Users have the option to split the data by *N*-fold cross-validation, where the model is trained on the *N* − 1-fold data and evaluated on the remaining 1-fold data.

### Pathway-level analysis

The selected pathway features from classification or prognosis prediction can be visualized with the Pathview R package [34]. Currently, any KEGG pathway can be used as the input to render pathway graphs. The top pathways are selected with the featureSelection() function in Lilikoi. Additionally, if there are corresponding gene expression profiles, they can be integrated with metabolites in Pathview.

The relationship between pathway and the metabolites in that particular pathway can be analyzed by single-variate regression. The metabolites that are significantly associated with the pathway are displayed as bar graphs and top tables. All pathway features and their significantly associated metabolites are visualized by a bipartite graph with Cytoscape style. Cytoscape modules are imported in Lilikoi by the RCy3 R package [35].

## Results

### Overview of updated functionalities in Lilikoi v2.0

The Lilikoi v2.0 package is a significant upgrade of the previous version. It keeps all 4 modules in the original Lilikoi v1 package: feature mapper, dimension transformer, feature selector, and classification predictor [2]. However, given the recent applications of deep learning in the metabolomics and other genomics fields [3–8], it is important to enable metabolomics researchers to investigate such new approaches. We thus implemented deep learning as a new method in the classification module. Moreover, metabolomics data have the potential to be prognosis markers [10]; however, at present it is rare for a metabolomics data analysis workflow to be available to handle this issue. We herein implemented multiple methods for prognosis prediction, including Cox-PH model and Cox-nnet, a neural network–based model [5]. Additionally, we augmented the pathway-based metabolomics analysis with metabolite-pathway relationship analysis and pathway visualization. Finally, we also include additional preprocessing methods for metabolomics data analysis (e.g., normalization, imputation) and tools for exploratory data analysis (e.g., PCA, t-SNE, and SOV analysis).

**Figure 1:**
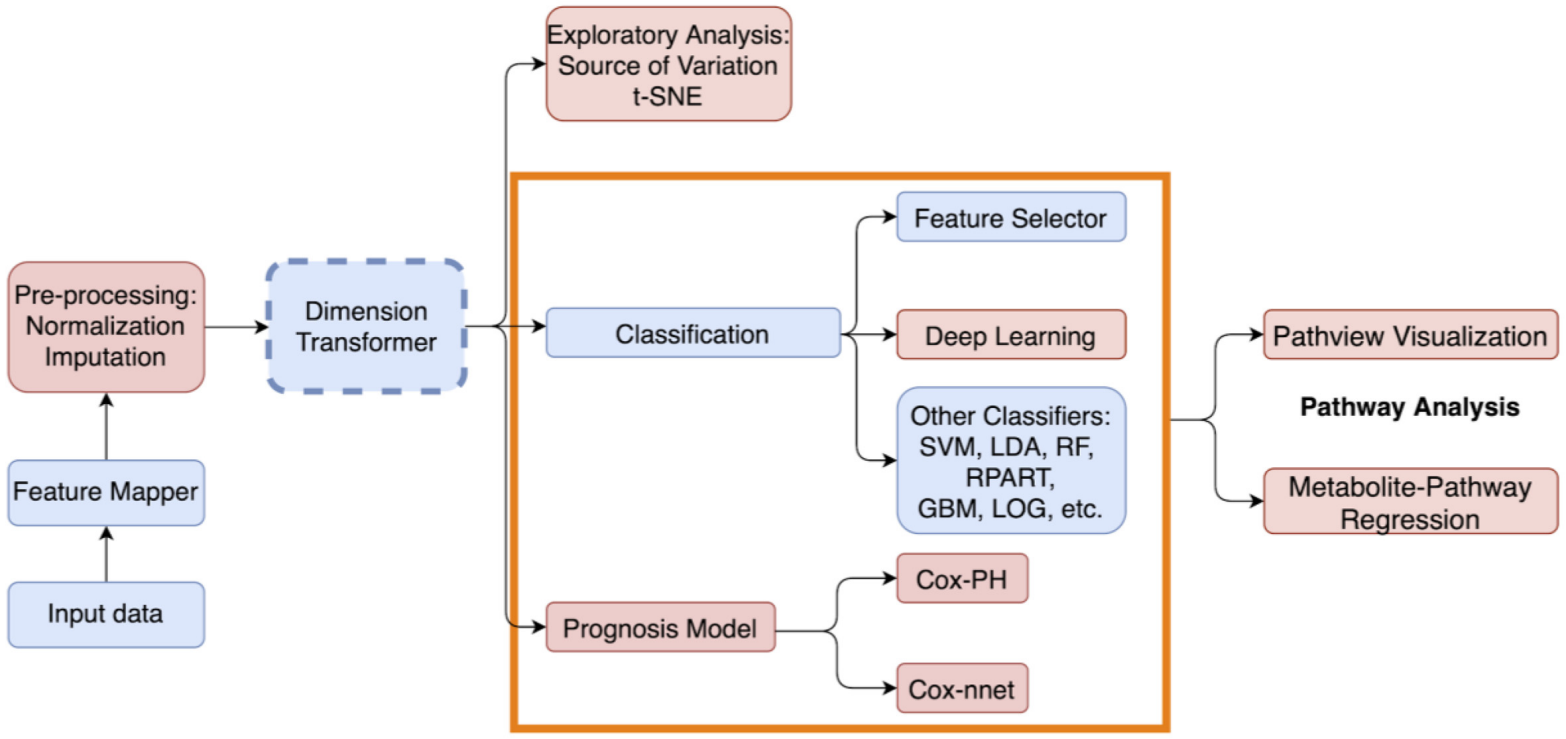
The workflow of the Lilikoi v2.0 package. Lilikoi v2.0 is composed of 7 modules: feature mapper, preprocessing, dimension transformer, exploratory analysis, classification, prognosis model, and pathway analysis. Input data require metabolomics data matrix, and 1 column of a categorical variable to specify case/control status for each subject (for classification), or survival information (for prognosis analysis). Feature mapping converts metabolite names to standardized metabolic IDs (e.g., Human Metabolome Database IDs) and then transforms them into pathway names. Preprocessing enables 3 normalization methods (standard, quantile, and median-fold) and 1 knn imputation method. The red boxes are new functionalities added to Lilikoi v2.0. Blue boxes are pre-existing modules in Lilikoi v1. Dashed box indicates an optional step.

Importantly, Lilikoi v2.0 has added the following new functionalities (indicated by red boxes in Fig. 1). A preprocessing module is added for the initial steps, where normalization and imputation are considered. A new exploratory data analysis module is also added, to enable dimension reduction analysis (PCA or t-SNE) and SOV. The classification module is amended with the new deep learning method, along with the previously implemented machine learning methods. Additionally, a new prognosis module is introduced in this version, where the Cox-PH method and a new neural network–based Cox-nnet method are implemented. Downstream analysis and interpretation of pathways is also a new add-on feature, where visualization and metabolite-pathway regression are available.

### Data preprocessing and exploratory analysis

For data preprocessing, we added normalization and imputation methods. Three normalization methods (standard, quantile, and median-fold) are implemented, with median-fold normalization as the default method. For imputation of missing values, knn is the default method.

Unsupervised exploratory analysis is an important step to better elucidate the pattern in metabolomics data, as well as the metabolomics-phenotype relationship. To enable this, Lilikoi v2.0 added PCA and t-SNE plots that help users to visualize high-dimensional metabolomics data. PCA reduces the dataset dimensions by determining the linearly independent dimensions based on the eigenvalues and eigenvectors of the covariance matrix to represent the data. Different from the linear dimension reduction of PCA, t-SNE maps the high-dimensional data onto a low-dimensional space via a non-linear algorithm.

To investigate the metabolomics-phenotype data relationship, Lilikoi v2.0 has added the source of variation analysis between confounders and metabolomics data, based on ANOVA tests [18]. Any clinical confounder with F-score bigger than the error term, whose F-score is 1, needs to be adjusted for in differential metabolite tests, when using other clinical variable(s) for grouping.

### Deep learning–enabled classification module

The deep learning–enabled classification module is one of the highlighted functionalities of Lilikoi v2.0. The deep learning framework uses the same dataset and adopts the same architecture as previously described [9]. The objective is to distinguish the 204 ER+ samples from the 67 ER− samples. We split the data in a roughly 4:1 ratio into training and test data, with 10-fold cross-validation in the training data. We repeated this process 10 times randomly, to obtain averaged metrics.

**Figure 2:**
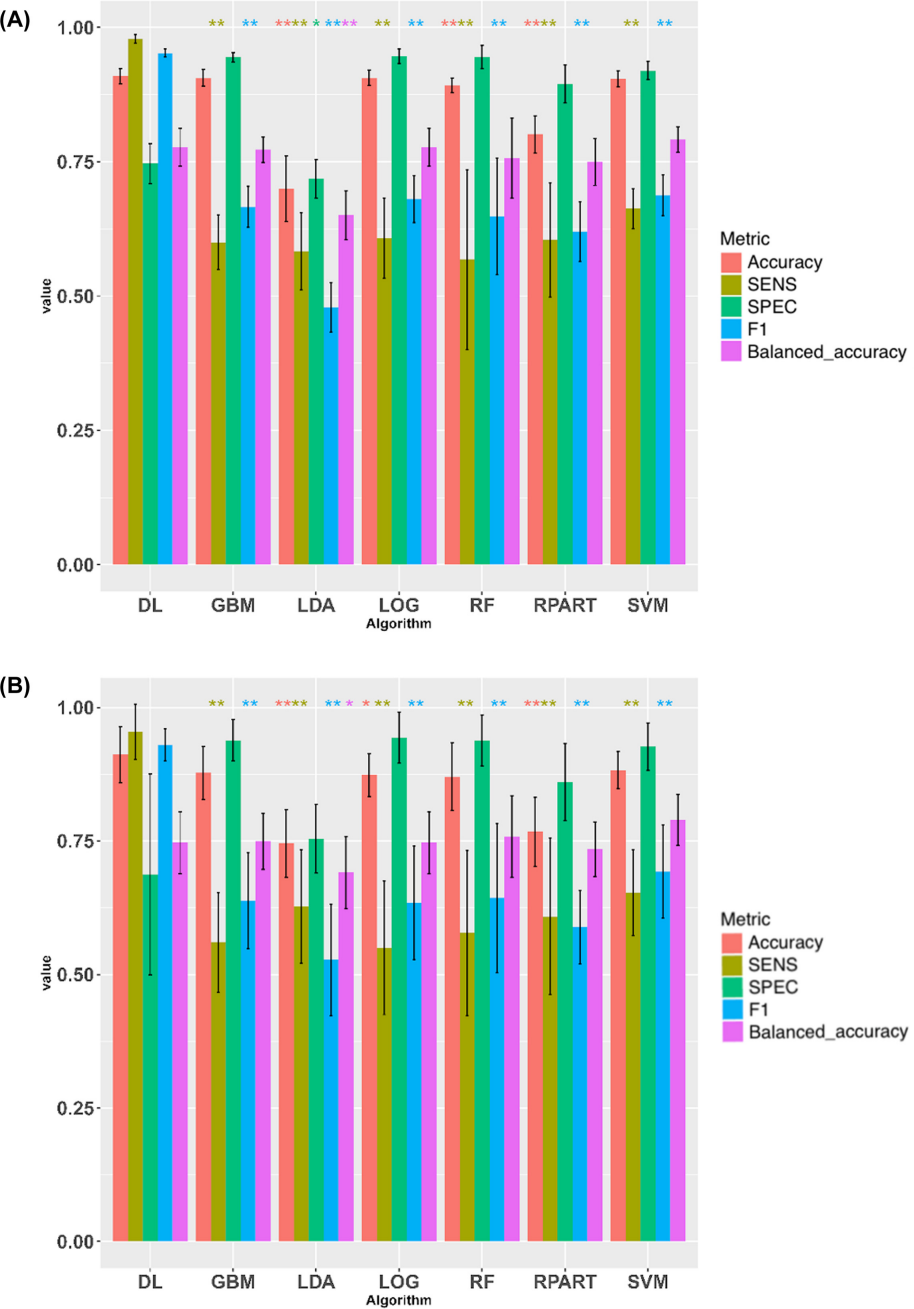
Model evaluation on deep learning (DL) and other machine learning techniques. (A) Metrics on training datasets; (B) metrics on test datasets. GBM: generalized boosted model; LDA: linear discriminant analysis; LOG: logistic regression; RF: random forest; RPART: recursive partitioning and regression analysis; SVM: support vector machine. *P < 0.05 (1-tail t-test) compared to the same metric in DL; **P < 0.01 (1-tail t-test); Accuracy, measures how well the model distinguishes between classes. Sensitivity (SEN) measures the capability of a model to correctly identify cases or diseases. Specificity (SPEC) measures the capability of a model to correctly identify controls or normal status. F1 statistic measures the accuracy of a model. Balanced accuracy is the mean of specificity and sensitivity, a good metric to consider when the sample sizes in cases and controls are not balanced.

We used the metabolite features as the inputs for deep learning–based classification, along with other popular methods: LDA, SVM, RF, RPART, LOG, and GBM (Methods). As shown in Fig. 2A and Table 1, deep learning on average performs the best overall in the training data, with a significantly higher F1 statistic value (0.95) and sensitivity (0.98) than all other methods. The F1 statistic is a good unbiased metric given the unbalanced samples in the ER+ and ER− classes. However, the specificity (0.75) in the training dataset is second to the lowest (SPEC of LDA = 0.72). The advantage of deep learning is more pronounced in the test dataset (Fig. 2B and Table 1), where it achieves the highest values in Accuracy = 0.91, SEN = 0.95, and F1 statistic = 0.93. Again the specificity is lower than other methods (0.69), probably due to the size of the samples. As a word of caution, the computation time to run the deep learning method is significantly longer than other machine learning methods, and it is only beneficial when the sample size is moderate (on the order of hundreds).

**Table 1:** Performance of classification models on training and reserved test dataset. Boldface signifies that DL method is statistically significantly better in the metric, compared to other methods.

Dataset	Algorithm	Accuracy	SENS	SPEC	F1 Statistic	Balanced accuracy	Computing time/run (sec)
Training	DL	**0.909**	**0.978**	0.747	**0.952**	0.777	570.68
	GBM	0.906	0.600	0.945	0.666	0.772	8.291
	LDA	0.700	0.583	0.718	0.478	0.651	3.118
	LOG	0.906	0.608	0.946	0.681	0.777	5.394
	RF	0.892	0.568	0.946	0.648	0.757	21.340
	RPART	0.801	0.605	0.895	0.620	0.750	3.525
	SVM	0.905	0.663	0.920	0.688	0.791	4.941
Testing	DL	**0.912**	**0.954**	0.688	**0.930**	0.747	1.844
	GBM	0.878	0.560	0.939	0.639	0.749	0.0152
	LDA	0.745	0.627	0.754	0.527	0.691	0.0149
	LOG	0.873	0.550	0.943	0.634	0.747	0.0184
	RF	0.870	0.578	0.938	0.643	0.758	0.0181
	RPART	0.767	0.609	0.861	0.589	0.735	0.0257
	SVM	0.883	0.653	0.927	0.693	0.790	0.0218

### Prognosis prediction

**Figure 3:**
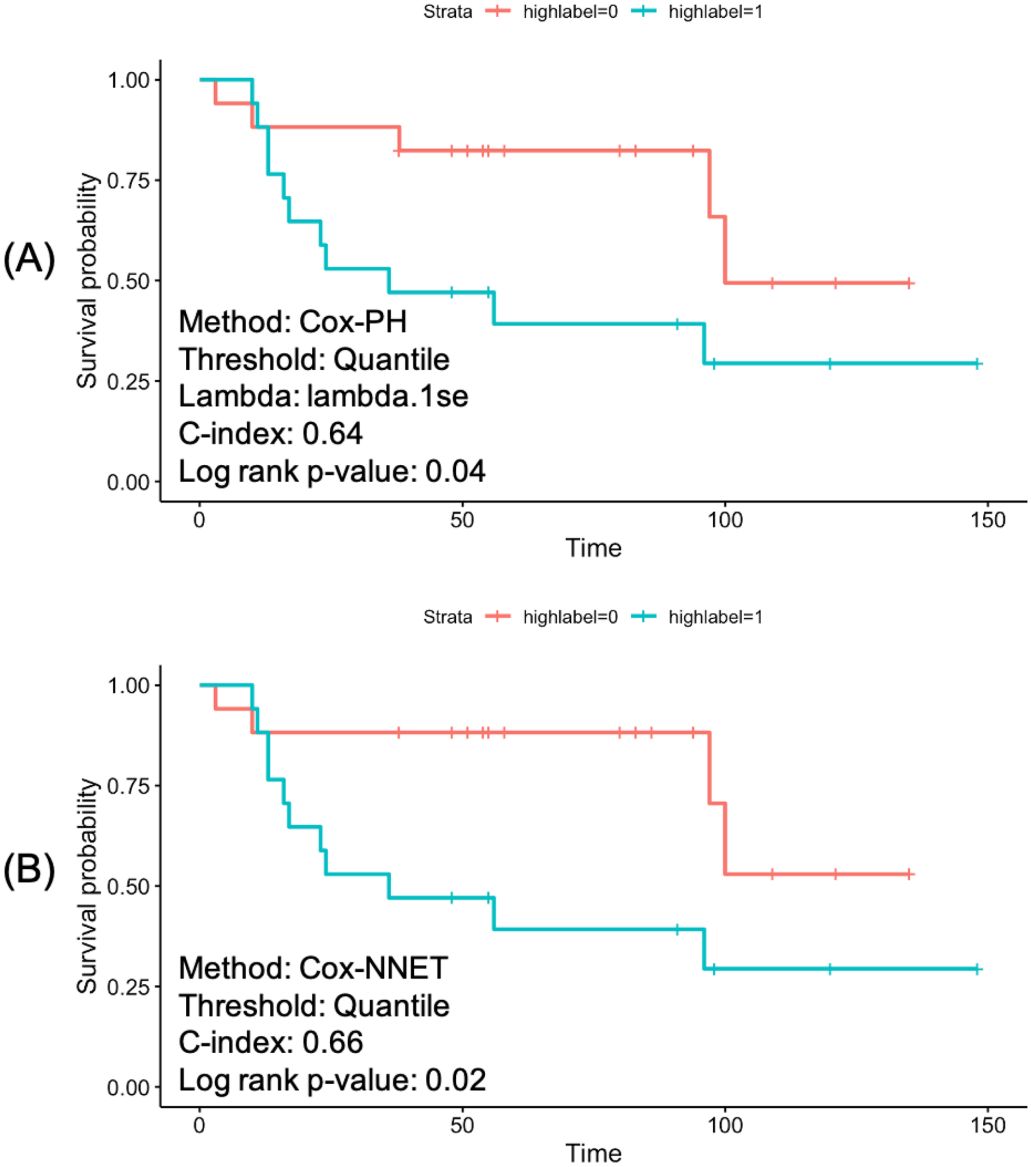
Comparison of Kaplan-Meier curves resulting from Cox-PH and Cox-nnet. The samples are dichotomized into 2 risk groups by the first quantile of the prognosis index (PI) score. (A) Cox-PH model. (B) Cox-nnet model with 3-layer neural network: 1 input layer, 1 fully connected hidden layer, and the output layer.

Deep learning–enabled prognosis prediction is another of the unique functionalities of Lilikoi v2.0, compared to other metabolomics analysis packages and toolkits. To demonstrate prognosis analysis, we used the NCI dataset as described in Methods. As the unique feature of Lilikoi is pathway-level modeling, the metabolites intensity data are first transformed to pathway-level data matrix (see Methods). Penalized survival analysis using Cox-PH model and Cox-nnet were conducted. For Cox-PH regression, L2 norm (Ridge) penalization was applied to select featured pathways. After fitting, the PI was used to separate the patients into the high-risk vs low-risk groups using the first quantile of PI as the threshold. As shown by the Kaplan-Meier curves in Fig. 3, the Cox-PH model yields a C-index value of 0.64 and log-rank *P*-value of 0.04 (Fig. 3A); the Cox-nnet model yields slightly better results, with a C-index value of 0.66 and log-rank *P*-value of 0.02 (Fig. 3B).

### Pathway downstream analysis

**Figure 4:**
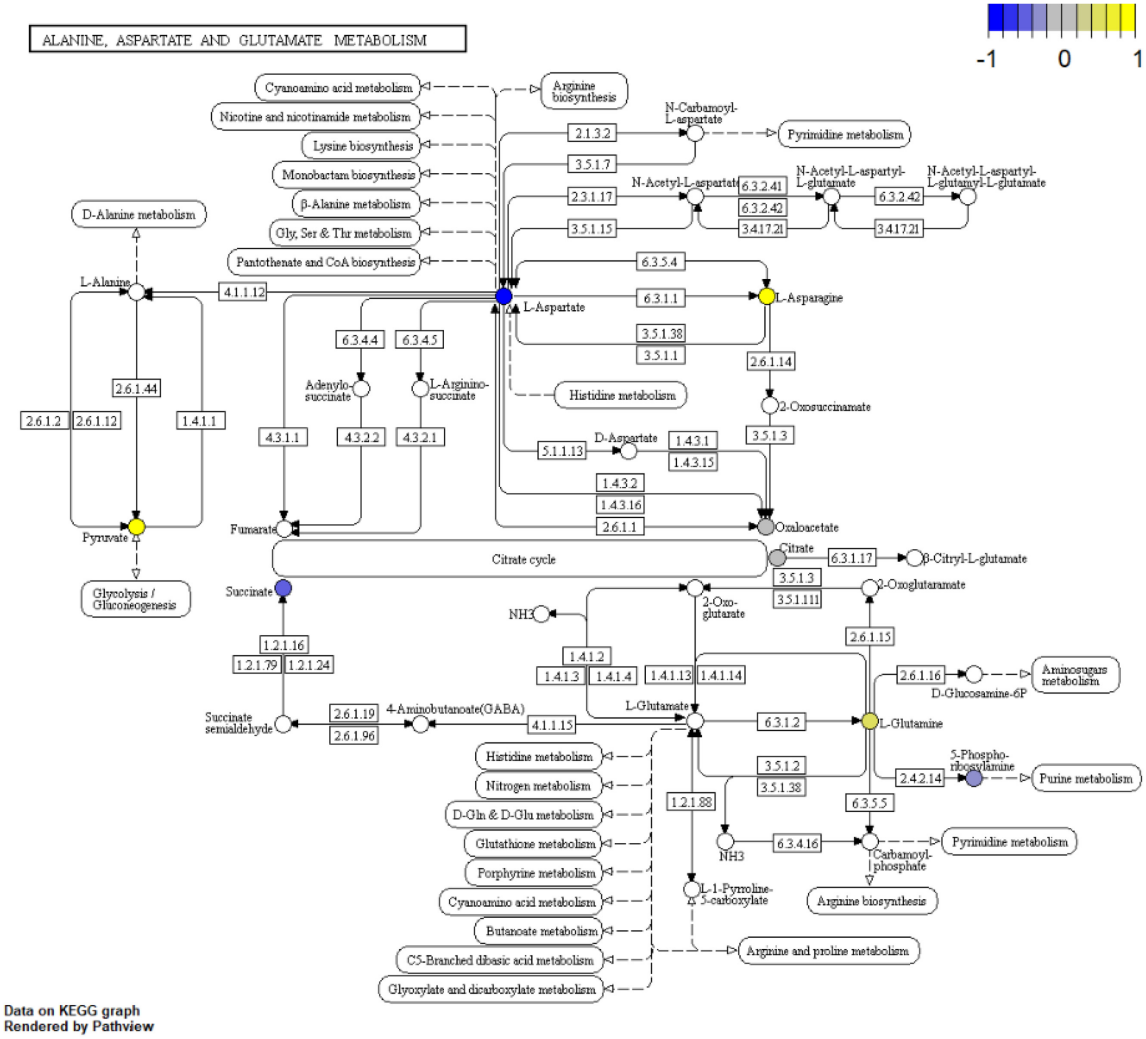
Pathway visualization: alanine aspartate and glutamate metabolism pathway. Color scheme is based on log_2_-transformed ratio of the mean values of ER− samples over ER+ samples. The pathway rendering was done by the Pathview R package.

We used the metabolite expression information in the aforementioned workbench breast cancer dataset PR000284 as the cpd.data input of the pathview function. According to our featureSelection results, alanine aspartate and glutamate metabolism is one of the top pathways for metabolite data. Therefore, we demonstrate the pathway visualization, based on the Pathview R package, using “alanine aspartate and glutamate metabolism pathway” (Fig. 4). As shown in Fig. 4, 6 metabolites in this pathway have intensities. Asparagine has increased levels in patients with ER− disease, due to the conversion from its substrate aspartate, which is reduced in patients with ER− disease. The reduction of aspartate in patients with ER− disease is consistent with the previous observation [36].

**Figure 5:**
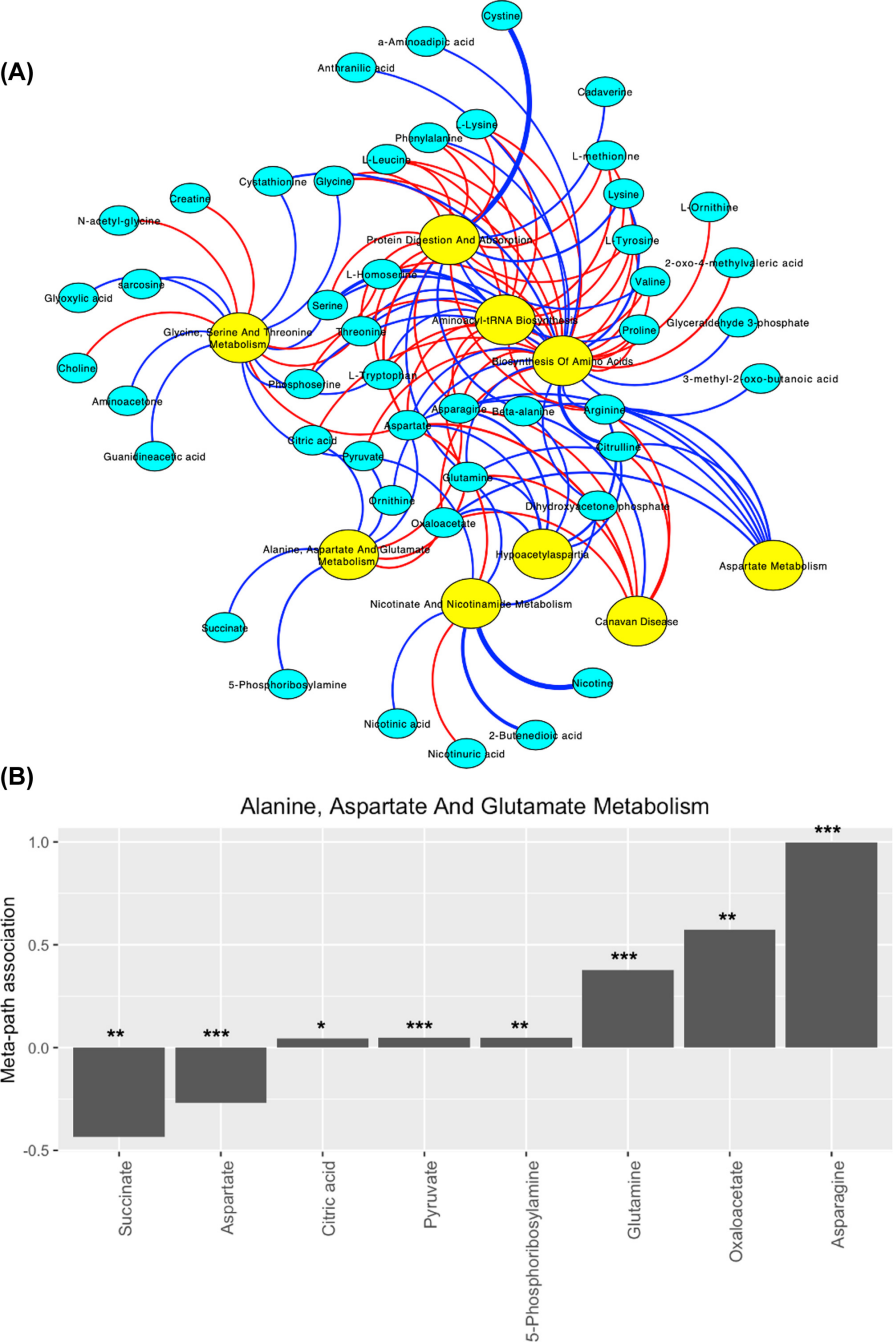
Metabolite-pathway relationship analysis. (A) Bipartite plot with top 10 pathways and corresponding metabolites. Cyan and yellow nodes indicate metabolites and pathways, respectively. Red and blue edges are negative (−) and positive (+) associations, respectively. Thicker edges indicate higher levels of association. (B) Bar plots of the relationship between the Alanine, Aspartate And Glutamate Metabolism pathway and its corresponding metabolites. *P < 0.05; **P < 0.01; ***P < 0.001.

It is important to link the significant metabolites that contribute to the pathway features. For this, single-variate regressions between metabolites and pathways are conducted, with the workbench dataset with 207 plasma samples (126 breast cancer cases and 81 control cases). The regression results (Fig. 5) can be visualized by the partite graph, where the yellow nodes represent pathway features and the cyan nodes are metabolites significantly (*P* < 0.05) associated with the pathways, showing how each metabolite contributes to the selected pathways. The generic term “metabolic pathways” is associated with the largest number (86) of metabolites. Among them, isopentenyl pyrophosphate has the most weight on the edge. Many pathways related to amino acid synthesis and metabolism are highlighted. Users can also elect to examine the metabolites within a particular pathway, by individual bar graphs. As an example, we show the metabolites that are associated with “alanine aspartate and glutamate metabolism pathway” (Fig. 5B). Citric acid, pyruvate, 5-phosphoribosylamine, glutamine, oxaloacetate, and asparagine all significantly (*P* < 0.05) increased in patients with ER− disease, with coefficients of 0.043, 0.046, 0.049, 0.378, 0.575, and 0.997 from single-variate linear regressions; on the other hand, succinate and aspartate have opposite significant decreases, with coefficients of −0.435 and −0.269. Additional bar graphs showing relationships of metabolites and all top 10 pathways are in Supplementary Fig. S1.

## Discussion and Conclusions

Here we report the upgrade of Lilikoi v2.0, a new deep learning–enabled, personalized pathway-based package for diagnosis and prognosis predictions using metabolomics data. The new version of Lilikoi added many new modules, including data preprocessing, exploratory analysis, deep learning, prognosis prediction, and visualization. Building on the previous work on pathway-based modeling and prediction, Lilikoi v2.0 allows much better exploration of pathway-based analysis using various modern analytics methods for classification and survival analysis, including deep learning implementation. Such an endeavor sets Lilikoi v2.0 apart from other more conventional metabolomics analysis packages [37–39]. One of the closest comprehensive packages is MetaboAnalystR [40]. Some functions are similar between the 2 tools, such as classification using caret packages. However, there are some very significant differences between the two, such as the aforementioned functionalities. On the other hand, MetaboAnalystR provides other functionalities, such as time-series analysis, power analysis, and network explorer, which Lilikoi does not have yet.

Some practical challenges still exist, leaving room for the future development of Lilikoi. For example, the mapping rate of metabolites and pathways can be further improved, by using better matching algorithms. Also, the current best classification model in Lilikoi is determined by users. We would like to automatically recommend the best classification model for users. This will be dependent on training a large set of metabolomics datasets for benchmarking, beyond of the scope of this report. Despite this, we recommend that users pay more attention to the machine learning methods that are less prone to overfitting, such as RF, given the fact that the majority of the datasets have moderate sample size (on the order of hundreds). The comparison between deep learning and other machine learning methods shows the advantages of increased accuracy of the deep learning method. However, such benefit is achieved at the cost of computation time. Moreover, the higher performance of the deep learning method is conditional on the sample sizes. Deep learning is superior when the patient size is at least a few hundred. Moreover, given the limited number of annotated metabolites (on the order of hundreds) in the assays, the pathway visualization is sparse, based on metabolites alone. Integration between metabolomics and other genomics data types is helpful to fill in the missing information, especially with the aid of deep learning and machine learning ensemble tools, such as DeepProg models that have been developed by us and others [3,4,8,41].

## Availability of Source Code and Requirements

Project name: Lilikoi Project home page: https://github.com/lanagarmire/lilikoi2

Operating system(s): Windows and macOS Programming language: e.g., R

Other requirements: e.g., R ≥ 3.5.0

Dependencies: car, caret, dplyr, gbm, ggplot2, glmnet, h2o, impute, infotheo, limma, M3C, Metrics, MLmetrics, parallel, pathifier, pathview, plyr, preprocessCore, pROC, RCy3, reticulate, reshape, RWeka, scales, stringr, survminer, survival

License: GPL-2

Lilikoi v2.0 source code with documentation and scripts to run testing data are available at https://github.com/lanagarmire/lilikoi2. Lilikoi v2.0 R package has been submitted to the CRAN team, and upon acceptance, it will be expected to be available at https://cran.r-project.org/web/packages/lilikoi/index.html.

## Data Availability

Snapshots of our code and data further supporting this work can be openly found in the GigaScience repository, GigaDB [42].

## Abbreviations

ANOVA: analysis of variance; Cox-PH: Cox–proportional hazards; CPU: central processing unit; ER+: estrogen receptor positive; NCI: National Cancer Institute; GBM: generalized boosted model; KEGG: Kyoto Encyclopedia of Genes and Genomes; knn: *k*-nearest neighbors; LDA: linear discriminant analysis; LOG: logistic regression; PCA: principal component analysis; PDS: pathway dysregulation score; PI: prognosis index; RAM: random access memory; RF: random forest; RPART: recursive partitioning and regression analysis; SEN: sensitivity; SOV: source of variation analysis; SPEC: specificity; SVM: support vector machine; t-SNE: t-distributed stochastic neighbor embedding.

## Competing Interests

The authors declare that they have no competing interests.

## Funding

This research was supported by grants K01ES025434 awarded by NIEHS through funds provided by the trans-NIH Big Data to Knowledge (BD2K) initiative (http://datascience.nih.gov/bd2k), R01 LM012373 and R01 LM012907 awarded by NLM, and R01 HD084633 awarded by NICHD to L.X.G.

## Authors' Contributions

L.X.G. envisioned and supervised the project. X.F. and Y.L. coded and analysed data, with help from Z.R., Y.D., and Q.H. X.F. and Z.R. wrote package documentation. All authors have read and approved the manuscript.

## Supplementary Material

giaa162_GIGA-D-20-00214_Original_Submission

giaa162_GIGA-D-20-00214_Revision_1

giaa162_GIGA-D-20-00214_Revision_2

giaa162_Response_to_Reviewer_Comments_Original_Submission

giaa162_Response_to_Reviewer_Comments_Revision_1

giaa162_Reviewer_1_Report_Original_Submission Biswapriya Misra -- 8/15/2020 Reviewed

giaa162_Reviewer_1_Report_Revision_1 Biswapriya Misra -- 11/19/2020 Reviewed

giaa162_Reviewer_2_Report_Original_Submission Zhibo Yang -- 8/23/2020 Reviewed

giaa162_Supplemental_Files
