# Supplementary material for: Lilikoi V2.0: a deep learning–enabled, personalized pathway-based R package for diagnosis and prognosis predictions using metabolomics data
Source: Gigascience. 2021 Jan 23;10(1):giaa162. doi: 10.1093/gigascience/giaa162 (PMC7825009; doi:10.1093/gigascience/giaa162)
Supplement: giaa162_GIGA-D-20-00214_Revision_1 [file giaa162_giga-d-20-00214_revision_1.pdf]

GigaScience

Lilikoi V2.0: a deep-learning enabled, personalized pathway-based R package for diagnosis and prognosis predictions using metabolomics data

--Manuscript Draft--

**Manuscript Number:**	GIGA-D-20-00214R1
**Full Title:**	Lilikoi V2.0: a deep-learning enabled, personalized pathway-based R package for diagnosis and prognosis predictions using metabolomics data
**Article Type:**	Research
**Funding Information:**	National Institute of Environmental Health Sciences (K01ES025434) — Dr Lana Garmire<br>Nancy Lurie Marks Family Foundation (R01 LM012373) — Dr Lana Garmire<br>Nancy Lurie Marks Family Foundation (R01 LM012907) — Dr Lana Garmire<br>National Institute of Child Health and Human Development (R01 HD084633) — Dr Lana Garmire
**Abstract:**	Previously we developed Lilikoi , a personalized pathway-based method to classify diseases using metabolomics data. Given the new trends of computation in the metabolomics field, here we report the next version of Lilikoi as a significant upgrade. The new Lilikoi v2.0 R package has implemented a deep-learning method for classification, in addition to popular machine learning methods. It also has several new modules, including the most significant addition of prognosis prediction, implemented by Cox-PH model and the deep-learning based Cox-nnet model. Additionally, Lilikoi v2.0 supports data preprocessing, exploratory analysis, pathway visualization and metabolite-pathway regression. In summary, Lilikoi v2.0 is a modern, comprehensive package to enable metabolomics analysis in R programming environment.
**Corresponding Author:**	Lana Garmire<br><br>UNITED STATES
**Corresponding Author Secondary Information:**	
**Corresponding Author's Institution:**	
**Corresponding Author's Secondary Institution:**	
**First Author:**	Xinying Fang
**First Author Secondary Information:**	
**Order of Authors:**	Xinying Fang<br>Yu Liu<br>Zhijie Ren<br>Yuheng Du<br>Qianhui Huang<br>Lana Garmire
**Order of Authors Secondary Information:**	
**Response to Reviewers:**	Answers to Reviewers:<br><br>Reviewer #1: The manuscript with ID GIGA-D-20-00214, entitled "Lilikoi V2.0: a deep-learning enabled, personalized pathway-based R package for diagnosis and prognosis predictions using metabolomics data" submitted by Xinying Fang; Yu Liu; Zhijie Ren; Yuheng Du; Qianhui Huang; Lana Garmire et al. to the journal GigaScience introduces

Powered by Editorial Manager® and ProduXion Manager® from Aries Systems Corporation

the version 2 of their DL -enabled metabolomic pathway analysis package Lilikoi. In deed innovative and interesting approach. Indeed a very important and timely topic. However, the following comments / suggestions/ advice are provided to help the authors improve on the currently submitted version of the manuscript only.

Critique and scope to improve
A table showing the comparison against currently popular pathway analysis tools and Lilikoi V1, for metabolomics-scale data, their in put output, ease of use, time of analysis, strengths and short comings, visualization types, statistical rigor, pre-processing capabilities, dependencies, would aid the reader to capture the current state of the art in the field.

Thank you for the suggestion. As far as we know, Lilikoi is the first package that offers personalized (or individualized) pathway scores, making it very distinct from other popular metabolomics enrichment analysis tools. Another earlier paper by Marco-Ramell etc [1] had done comprehensive comparisons on 13 methods, which were different variations of Fisher's exact tests or hypergeometric tests. We therefore refer the readers to this benchmark study to avoid repetitions.
Refrenece: [1]. Marco-Ramell A, Palau-Rodriguez M, Alay A, Tulipani S, Urpi-Sarda M, Sanchez-Pla A, et al. Evaluation and comparison of bioinformatic tools for the enrichment analysis of metabolomics data. BMC Bioinformatics [Internet]. BioMed Central; 2018 [cited 2020 Nov 17];19. Available from: https://www.ncbi.nlm.nih.gov/pmc/articles/PMC5749025/

This reviewer faced issues installing lilikoi upfront:
"Warning message:
package 'lilikoi' is not available (for R version 4.0.2)"
and also
"Warning message:
package 'lilikoi' is not available (for R version 3.5.2)"
These may be resolved by the authors too.
The dependencies on Python, R and other packages are not clearly mentioned/ listed in the MS.
Our apologies. The link to Lilikoi was not approved by CRAN team at the time of last review, but it is updated and available on CRAN now: https://cran.r-project.org/web/packages/lilikoi/index.html. We also mentioned clearly the dependencies in the manuscript revision (Method section).

The tool will greatly benefit with PDF stepwise and detailed manual which is clearly missing.
Thank you for the suggestion. We attach the manual as the supplementary material. It is also available in CRAN R package.

Even using the Shiny tool (https://lilikoi.shinyapps.io/lilikoi/) specified "sample data" leads to error messages such as "An error has occurred. Check your logs or contact the app author for clarification." Under : "feature mapping" tab. Plus, the next steps on Shinny are extremely slow, and are time-intensive in this reviewers experience. A lot of scope to improve.
Our apologies. The Shiny app was for the older version. Users are now strongly encouraged to use/download Lilikoi v2 as the R package and on the new Github website for Lilikoi v2.

Too elaborate conclusion, keep it crisp and clear with a strong message. Unsure how NLP will improve any of these.
Thank you for the suggestion. We have now removed NLP mentioning in the revision.

Authors should also come up with recommendations that would guide the users of their tool, for instance:
-which of the DL, GBM, LDA, LOG, RF, RPART, SVM perform optimally for LC-MS vs GC-MS vs NMR data?
Thank you for the good question. It is very difficult, if ever possible, to make a generalized recommendation, as the results are really data and sample size dependent. As a rule of thumb, we do recommend users in the revision, to pay

Powered by Editorial Manager® and ProduXion Manager® from Aries Systems Corporation

attentions to machine learning methods that are less prone to overfitting (such as random forest, or RF)

-Which options are "Default" before reaching to the prognosis model from the input data?
Thank you for the questions. We have clarified these in the revision, whenever default is needed.
For Cox-nnet, the default optimization method is the standard gradient descent.
For Cox-PH, the default lambda parameter for prediction in Cox-PH is lambda.1se. The default penalization method alpha is 1, which is the Lasso penalization.

Describe what kind of computational resources one user needs to perform all these analysis, and how many datasets can Lilikoi v2 handle and in what time? Any bench marking done against any other comparable tools?
Version of R: 3.5.1 The users can run the job on the desktop or on the server. Deep learning is more time-consuming on the desktop with specs of macOS with a processor of 2.6 GHz 6-Core Intel Core i7 and memory of 16 GB 2667 MHz DDR4. It takes about 15 mins for each run.
Additionally, we have listed the running time for each machine-learning method as an additional field in Table 1.
The closest comprehensive packages that we know is MetaboAnalystR. Some functions are similar between the two, such as classification using caret packages, however, there are some very significant differences between the two as well, making it impossible to benchmarking, For example, deep-learning modules are unique and only available to our package, to our knowledge. We also have modules for prognosis prediction, which is unique. The individualized pathway calculation is our new offering. On the other hand, MetaboAnalystR provides other functionalities, such as time-series analysis, power analysis, and network explorer. We added the comparisons between them in the discussion section.

Terms such as "pathway deregulation" and " "pathway dysregulation" need to be defined, contextualized for the readers.
Apologies. The correct term should be "pathway dysregulation". We corrected in the revision, and added the definition: “PDS is a normalized score between [0,1] that measures the degree of dysregulation of a pathway relative to the norm (controls)”.

Table 1, for some metrics why does the "testing" has a better score than the "training" data set?
Thank you for the comment. The very small differences (0.0x) between testing and training data are presumably due to the randomness in the seeds that were used to split the training and testing data. We did statistical tests among the runs, and found that none of the differences in the metrics are significant.

The authors have smartly used openly available metabolomics datasets for the tool development/ validation which is appreciable.In the "Datasets" section specify as to which platforms, LC-MS, GC-MS were used to generate the original datasets besides the accession numbers will help readers relating with the platform capabilities and data structure/size.
Thank you for your encouragement. We now have revised the Datasets section of the manuscript to added the platforms and the number of metabolites, whenever they were reported by the original publications (in the first 2 datasets).

Unsure why accessing the CRAN package this reviewer gets these messages:
"Package 'lilikoi' was removed from the CRAN repository.
Formerly available versions can be obtained from the archive.
Archived on 2020-07-22 for policy violation.
Please use the canonical form https://CRAN.R-project.org/package=lilikoi to link to this page."
Sorry, this problem is now fixed and the package is available (at the time of this revision submission). We had some issue with lilikoi v1 maintenance due to the departure of a previous group member, we have now designated the PI as the contacting person responsible for the active maintenance of the package.

Table 1. Is this is the sole "performance" defining analysis? What's the time-taken for

	analysis for each of these on the 3 datasets used?<br>Thank you for the good question! We have added the running time as additional performance metric in Table 1.<br><br>Figure 1 lacks details: Should specify what "input data" format, what does "Feature mapper" do?, which Normalization methods to choose, which imputation methods apply. Without granular informations it hard to find usefulness of the workflow.<br>Thank you for the comment. We have added more explanation in the figure 1, particularly, we added:<br>“Input data requires metabolomics data matrix, and one column of a categorical variable to specify case/control status for each subject (for classification), or survival information (for prognosis analysis). Feature mapping converts metabolite names to standardized metabolic IDs (e.g. HMDB IDs) and then transforms them into pathway names. Pre-processing enables three normalization methods (standard normalization, quantile normalization and median-fold normalization) and one knn imputation method.”<br><br>Also, Figure 1 indicates that "classification and "prognosis models" do not interact?<br>Yes, They are treated as two different types of models: classification models vs. survival models, since the meta data are different. Classification needs class labels, and survival model needs time-to-event survival information.<br><br>Figure 2A, B are not well explained in the text in terms of their significance and statistically important metrices.<br>Thank you for your suggestions. We have explained the metrics better in the figure legend:<br>“AUC, or accuracy, measures how the model can distinguish between classes. SEN, or Sensitivity, measures the capability of a model to correctly identify cases or diseases. SPEC, or Specificity, measures the capability of a model to correctly identify controls or normal status. F1-statistics measures the accuracy of a model. Balanced accuracy is the mean of specificity and sensitivity, a good metrics to consider when the sample sizes in cases and controls are not balanced.”<br><br><br>Figure 4 might be representative but the data is too sparse in terms of the # of metabolites mapped?<br>Yes, this is a general issue of metabolomics technology, we usually see 200-300 metabolites in a data set, and there are some missing metabolites (about 20%) in the pathways.<br><br>Figure 5 needs to improve, unreadable, too many over lapping nodes, can not read the metabolites/ pathways or have been cut out. Too many characters " ' " ' in those names that need a clean up. Legend with colour and node size, edge descriptions are missing too. Also, in the inset with the small bar graph, what's the Y-axis label ?<br>Thank you for the suggestions. We removed the characters “‘”. We modified the layout and the size for the nodes, and made metabolites/pathway names more clear..<br><br>The supplementary document "er_162" should provide the various metabolite Identifiers such as KEGG, Metlin, HMDB, SMILES, InChiKeys for future facilitation of studies on this dataset. Provide the list of qualifier/ quantifier ions, retention times (RTs) MS/MS spectral matrix (intensity and m/z) for aiding the readers.<br>Same comment as above for "JCI71180sd2_processed_tumor" and "plasma_breast_cancer" and "JCI71180sd2_processed_lilikoi"documents.<br>Thank you for the suggestions. We have added different IDs for metabolites in the new csv files. The “JCI71180sd2_processed_tumor” file is the tumor sample subset of “JCI71180sd2_processed_lilikoi” file, so we just uploaded the identifier file for the “JCI71180sd2_processed_lilikoi” file. The original datasets didn’t have M/Z score, so we couldn’t add them.<br><br>Moderate linguistic (English) corrections are desirable. Present version has some common, punctuation, tense, grammatical, typological sentence framing/ phrase construction issues which should be taken care by the authors.<br>Thanks. We did do additional rounds of proofreading to improve them.

Powered by Editorial Manager® and ProduXion Manager® from Aries Systems Corporation

	Please change and improve upon the conclusion to provide very specific and key take-home messages to the readers and should be only based on the findings and not extrapolations/ future aspects.<br>Thank you. We have improve the conclusion section with additional take-home messages from the results. We removed extrapolations and future aspects.<br><br>References are NOT OK and not well organized in terms of formats. Follow the journal format typically as instructed to the authors. Please be consistent.Check all the references one by one manually, so that everything cited in the text are also listed in the list of references.<br><br>Thank you. We have done reference checking more carefully.<br><br>If at all a revised version is submitted, then please make all changes in differently colored fonts (red/blue) or highlighted background (yellow) so that it would save the reviewer's time in finding the changes with lesser efforts and time.<br>We have used blue font in the revision, for the changes we made.<br><br>Please also incorporate all the answers/ discussions/ points raised by this reviewer into the manuscript rather than just explaining to this reviewer- in order to make the manuscript stronger than it is now.<br><br>We are including the point-to-point answers to reviewers, along with the revision.<br><br><br><br><br>Reviewer #2: The studies reported in this manuscript provide an updated version of Lilikoi. This new version software includes several new features, including deep-learning classification, prognosis prediction,<br>data preprocessing, and metabolite-pathway regression. A large amount of data was used for model training and testing. Compared with its previous version, this new version is capable of more comprehensive analysis. This manuscript has been well prepared. Some minor revisions are needed to improve its quality.<br><br>(1) Data from different types of samples, such as plasma and tissue, were used to train and test models, but it is unclear how they were correlated or integrated. Apparently, metabolites from plasma and tissues have different compositions and abundances.<br>Sorry for the confusion. The three data sets were used to address objectives in 3 different sections in the results. In each result section, the train and test dataset are subsetted from the same dataset.<br><br>(2) Prognosis prediction is an important new feature; however, it is unclear how metabolomics data and prognosis prediction methods were integrated. It seems that "The input parameters are event (eg. death), survival time and penalized covariates" is for the prediction methods, whereas the input from metabolites was not clearly described.<br>Thank you for the question. The input data are metabolite data as well as the time to event data. We have clarified this in revision (Fig 1 legend).<br><br>(3) Language issues. The following sentences need to be revised. "For imputation, we used k-nearest neighbors method…", in which the abbreviation "knn" is missing. "F-1 statistic is a good unbiased metric given the unbalanced samples in ER= and ER- classes" has a typo "=". "The reduction of aspartate in ER- patients is consistent with observation before."; it should be "previous observation". "metabolites significantly (p<0.05) associated with the pathways. show how each metabolite contributes to". "The generic term "metabolic pathways", is associated with the largest number of metabolites." In Conclusion, "Such endeavor sets Lilikoi apart from other more conventional metabolomics analysis packages"; it seemed that authors meant Lilikoi 2.0.<br>Thanks for the comments. We have done additional rounds of revision. We fixed the typos as the reviewer pointed out.
**Additional Information:**	

Powered by Editorial Manager® and ProduXion Manager® from Aries Systems Corporation

Question	Response
Are you submitting this manuscript to a special series or article collection?	No
**Experimental design and statistics**<br><br>Full details of the experimental design and statistical methods used should be given in the Methods section, as detailed in our Minimum Standards Reporting Checklist. Information essential to interpreting the data presented should be made available in the figure legends.<br><br>Have you included all the information requested in your manuscript?	Yes
**Resources**<br><br>A description of all resources used, including antibodies, cell lines, animals and software tools, with enough information to allow them to be uniquely identified, should be included in the Methods section. Authors are strongly encouraged to cite Research Resource Identifiers (RRIDs) for antibodies, model organisms and tools, where possible.<br><br>Have you included the information requested as detailed in our Minimum Standards Reporting Checklist?	Yes
**Availability of data and materials**<br><br>All datasets and code on which the conclusions of the paper rely must be either included in your submission or deposited in publicly available repositories (where available and ethically appropriate), referencing such data using a unique identifier in the references and in the "Availability of Data and Materials" section of your manuscript.	Yes

Have you have met the above requirement as detailed in our Minimum Standards Reporting Checklist?	

Powered by Editorial Manager® and ProduXion Manager® from Aries Systems Corporation

Manuscript

Click here to access/download;Manuscript;Copy of Lilikoi GigaScience version.docx

Lilikoi V2.0: a deep-learning enabled, personalized pathway-based R package for diagnosis and prognosis predictions using metabolomics data

Xinying Fang[1*], Yu Liu[2*], Zhijie Ren[3], Yuheng Du[1], Qianhui Huang[1], Lana X. Garmire[2$]

[1]Department of Biostatistics, School of Public Health, University of Michigan, Ann Arbor, MI, USA

[2]Department of Computational Medicine and Bioinformatics, University of Michigan, Ann Arbor, MI USA

[3]Department of Electric Engineering and Computer Science, University of Michigan, Ann Arbor, MI, USA

[*]These authors contributed equally to the work

$ corresponding author, email: lgarmire@med.umich.edu

ABSTRACT

Previously we developed *Lilikoi*, a personalized pathway-based method to classify diseases using metabolomics data. Given the new trends of computation in the metabolomics field, here we report the next version of *Lilikoi* as a significant upgrade. The new *Lilikoi* v2.0 R package has implemented a deep-learning method for classification, in addition to popular machine learning methods. It also has several new modules, including the most significant addition of prognosis prediction, implemented by Cox-PH model and the deep-learning based Cox-nnet model. Additionally, *Lilikoi* v2.0 supports data preprocessing, exploratory analysis, pathway visualization and metabolite-pathway regression. In summary, *Lilikoi* v2.0 is a modern, comprehensive package to enable metabolomics analysis in R programming environment.

KEYWORDS: classification, prognosis, survival, prediction, neural network, deep learning, modelling, Cox proportional hazards, metabolomics, pathway, visualization

INTRODUCTION

Metabolomics is an increasingly popular platform to systematically investigate metabolites as potential biomarkers for diseases [1]. With the rapid development in this field, data analysis is becoming a critical component to interpret and apply the results for translational and clinical research. However, currently the majority of metabolomics analysis workflows are provided as web-apps [1], limiting its adaptation by the bioinformatics community, and/or integration with other omics workflows in a programmable matter.

To address such needs, previously we developed *Lilikoi*, a personalized pathway-based method to classify diseases using metabolomics data [2]. Different from other metabolomics analysis packages, the personalized and pathway-based representation of metabolomics features is the highlight of *Lilikoi* version 1 (v1). *Lilikoi* v1 enables classifications using various machine learning methods. It has four modules: feature mapper, dimension transformer, feature selector, and classification predictor [2].

Here we report *Lilikoi* v2.0, as a significant upgrade for *Lilikoi* v1. The new mission is embarked by several recent trends or needs in the research community. Firstly, given the recent applications of deep-learning in the metabolomics and other genomics fields [3–9], it is important to enable metabolomics researchers to investigate such new approaches. We thus implemented a deep-learning neural network, as a new method in the classification module. Secondly, metabolomics have the potential to be prognosis markers [10], however, currently rarely metabolomics data analysis workflow is available for prognosis modeling and prediction. We herein implemented multiple methods for prognosis prediction, including Cox-Proportional Hazard (Cox-PH) model and *Cox-nnet*, a neural-network based model [5]. Thirdly, we augmented the pathway-based metabolomics analysis with metabolite-pathway regression and pathway visualization. Last but not least, we also include additional preprocessing methods for metabolomics data analysis (eg. normalization, imputation) and tools for exploratory data analysis (eg. PCA and t-SNE analysis, and source of variation analysis). In summary, *Lilikoi* v2.0 is a more mature, comprehensive and modern package to empower the metabolomics community.

METHODS

Datasets

Three breast cancer metabolomics datasets were used to demonstrate the new functionalities of *Lilikoi* v2.0. The first set was downloaded from the Metabolomics Workbench project ID PR000284 [11], which used 207 plasma samples (126 breast cancer cases and 81 control cases) with 227 metabolites from a previous study [2]. GC/MS and LC/MS metabolomics profiling were used to generate the dataset. The second metabolomics dataset is from a biobank at the Pathology Department of Charité Hospital, Berlin, Germany. It contains 162 metabolites from 271 breast cancer samples, where 204 samples are ER+ and 67 samples are ER- [12]. Metabolomics in this dataset were based on GC-TOFMS. The third dataset is shared by authors from an original National Cancer Institute (NCI) study, composed of 536 metabolites from 67 breast tumor samples and 65 tumor-adjacent noncancerous tissues [10]. In our analysis, we only used the 67 breast tumor samples for prognosis modeling.

Data preprocessing

For data preprocessing, we added normalization and imputation methods. Three normalization methods (standard normalization, quantile normalization and median-fold normalization) were implemented. We used the normalize.quantiles function in the *preprocessCore* package [13] to perform the Quantile normalization. For imputation, we used k-nearest neighbors (knn) method as the default method to impute missing values. The knn imputation was done by the impute.knn in the *impute* R package [14].

Exploratory analysis

Principal Component Analysis (PCA) is a feature selection technique [15]. It extracts the most important information in high-dimensional datasets. The t-SNE plot is a dimension reduction method to help users to visualize high-dimensional data [16] . We implemented the PCA and t-SNE plots in *Lilikoi v2.0* via the *M3C* package [17]. We also added the source of variation analysis (SOV) for

exploratory analysis, implemented by the Anova function in the *car* package [18]. SOV identifies the relationships between confounders and metabolomics data, based on ANOVA tests [19,20]. Any clinical variable with F-score bigger than the error term, whose F-score is 1, is deemed a confounder.

Metabolite to pathway level transformation

Most other pathway analysis tools for metabolomics data that use Fisher's exact test or hypergeometric test, and their performance was compared earlier [21]. Different from all these method, Lilikoi uses the *Pathifier* algorithm to perform the metabolites-pathway dimension transformation per sample [22] . For each pathway P in each patient i, a pathway dysregulation score (PDS) $D_P(i)$ between 0 and 1 is generated, based on the metabolites associated with this pathway. A larger PDS value represents a higher degree of dysregulation (larger deviation from the normal controls). As the result of the dimension transformation, a new pathway-level matrix is constructed, which can be used to substitute the original metabolomics profile matrix, for downstream classification or prognosis modeling.

Briefly, a PDS score $D_P(i)$ is calculated as the following: in the high-dimensional space d_P made of metabolite vectors (where each metabolite belongs to pathway P), all samples form a data cloud, where sample i is a data point x_i. The principle curve $S_{P'}$ in this space d_P is then computed using Hastie and Stuetzle's algorithm [23]. For each sample, the data point x_i is projected onto the principle curve $S_{P'}$. The dysregulation score $D_P(i)$ of sample i is then defined as the distance from the start of the principal curve to the projected point on this curve. More details of applications of *Pathifier* on biomarker studies (prognosis or diagnosis) can be found in earlier publications [2,24,25].

Deep learning for classification

The deep learning algorithm in *Lilikoi v2.0* is based on the *h2o* package [26–28]. It used a multi-layer neural network trained with stochastic gradient descent search to predict the diagnosis results. For the neural-network configuration, users are free to set parameters including activation function, hidden layer size, drop-out ratio, L1 and L2 regularization, size of one batch and adaptive learning rate decay factor. Users can also incorporate other control parameters like random discrete to optimize the hyperparameter setting to achieve the best deep learning performance.

Lilikoi *v2.0* supports users to run hyperparameter grid search on multiple deep learning models to achieve the best classification results. The activation functions are set as “Rectifier” or “Tanh”. Seven hiddenlayer configurations are pre-set for selections: 1 hidden layer setting (100 or 200 neurons), 2 hidden layer setting (10, 20 or 50 neurons for each layer), 3 hidden layers with 30 neurons for each, and 4 hidden layers with 25 neurons for each. The input dropout ratio options range from 0 to 0.9 with 0.1 increment. The number of global training samples per iteration is set to 0 or -2, where 0 means 1 epoch and -2 means the automatic value selected by the *h2o* package. The max number of times to iterate the whole dataset (epochs) is set as 500. The starting value of momentum is 0 or 0.5 (default 0, without hyperparameter grid search). The momentum damps the oscillation to achieve the optimal point and accelerates the iterations for faster convergence. The adaptive learning rate decay factor (rho) is 0.5 or 0.99 (default 0.99, without hyperparameter grid search). The quantile value (quantile_alpha value in *h2o*), when running quantile regression, is set between 0 and 1. , Quantile regression is similar to linear regression, but measures the conditional quantile rather than the conditional mean of the response variable. The threshold between quadratic and linear loss (huber_alpha value in *h2o*) is set between 0 to 1 (default 0.9). “RandomDiscrete” strategy is used to enable search on all combinations of the hyperparameters. As part of the automatic machine-learning training, the maximum number of models for each run is set to 100. The training steps stop if the misclassification values do not improve by 0.01 after 5 iterations. Score_duty_cycle, the frequency of computing validation metrics, is set to 0.025 in *lilikoi v2.0*, meaning that no more than 2.5% of the total training time should be used to build the validation metrics.

For the exemplary ER dataset, after grid search, the final hyperparameters for its deep learning model are set as the following: “Rectifier” activation function, four 25-neuron hidden layers, input dropout ratio 0, default training samples per iteration per *h2o* (value of -2), epoch value of 430.9, momentum starting value 0, a rho value of 0.99, a quantile regression value of 1, and a huber alpha value of 0, other hyperparameters including an L1 regularization value of 2.5e-5, and an L2 regularization value of 2.6e-5.

This deep learning algorithm is added in classification along with other 6 machine learning techniques previously implemented in *Lilikoi v1*, namely generalized boosted model (GBM), linear discriminant analysis (LDA), logistic regression (LOG), random forest (RF), recursive partitioning and regression analysis (RPART), support vector machine (SVM). Based on the data and sample size, users are free to choose which algorithms they would like to use. An n-fold cross validation (default n=10) is applied to avoid overfitting. Classification metrics such as Area Under the Curve (AUC), F1-statistic, balanced accuracy, sensitivity (SEN), and specificity (SPEC) are reported as bar plots.

The running time for each classification method is calculated with the Sys.time() function in R and measured using slurm job scheduler on a dedicated group computer server cluster (consisting of 4 nodes (Dell PowerEdge C6420) of 2 X Intel(R) Xeon(R) Gold 6154 CPU @ 3.00GHz, 192GB RAM). One processor and 50GB memory were reserved for each job.

Prognosis prediction

Lilikoi v2.0 enables prognosis prediction, at either the metabolite level using metabolite-sample matrix, or the pathway-level (after *pathifier* based pathway transformation) using pathway dysregulation score (PDS)-sample matrix. PDS is a normalized score between [0,1] that measures the degree of dysregulation of a pathway relative to the norm (controls). Currently two prognosis prediction methods are implemented: Cox-Proportional Hazards (Cox-PH) method [29] with penalization, and the neural-network based Cox-nnet method [5]. *Cox-PH* is a survival regression model developed by David Cox in 1972. The input parameters are event (eg. death), survival time and penalized covariates: alpha to determine which penalization method to use, and lambda (lambda.min or lambda.1se) for prediction. Penalization is achieved by Lasso, Ridge or Elastic net with the *glmnet* package [30]. The default lambda parameter for prediction in Cox-PH is lambda.1se. The default penalization method alpha is 1, which is the Lasso penalization.

Cox-nnet is based on the artificial neural network (ANN) framework with a default of two-layer neural network: a hidden layer and an output layer [31]. The output layer is fit to the Cox regression. *Lilikoi* v2.0 imports *Cox-nnet* originally written in Python, using the *reticulate* package.

The hazard function of the Cox-PH model is:

$$h(t|x_i) = h_0(t)exp(\theta_i)\text{with the log hazard ratio of } \theta_i = {x_i}^T\beta$$

with its partial likelihood cost function :

$$pl(\beta) = \Sigma_{C(i)=1}(\theta_i - log\Sigma_{t_i \geq t_j} exp(\theta_j))$$

The Cox-nnet expands the Cox-PH hazard function above as:

$$\theta_i = G(Wx_i + b)^T\beta,$$

where $x_i$is the output of the hidden layer, G is the activation function and W is the coefficient weight matrix between the input and hidden layer.

$$Cost(\beta, W) = pl(\beta, W) + \lambda(||\beta||_2 + ||W||_2)$$

In the demonstration NCI data, we applied cross validation on the training dataset to determine the optimal L2 regularization lambda parameter. Cox-nnet supports three gradient descent algorithms: standard gradient descent, nesterov gradient descent and momentum gradient descent. The default algorithm for Cox-nnet is the standard gradient descent. The Hyper-parameters can be set by users, including the gradient descent algorithm, initial learning rate, proportion of momentum, decrease of the learning rate, increase of the learning rate, number iterations between cost functions to determine increase or decrease of the learning rate, maximum number of iterations, stopping threshold, minimum number of iterations before stopping, number of iterations for new lowest cost before stopping and the random seed. The details can be found in the user manual.

The prognosis model is visualized by Kaplan-Meier curve plot, using the *survminer* package [32]. Samples are dichotomized into different risk groups by prognosis index (PI), the logarithm of hazard ratio of the prognosis model. Lilikoi v2.0 allows several approaches for dichotomization: median PI threshold, event/non-event ratio, and quantile PI threshold (samples with PIs under the 1st quantile as the low-risk group and those above the 3rd quantile as the high-risk group). The default dichotomous method in lilikoi v2.0 is median PI threshold.

The fitness of the models is evaluated by two metrics: C-index and log rank p-values. C-index is a goodness of fit measure of survival models [33]. A C-index of 1 indicates that the model is the best model for prediction and C-index = 0.5 means that the model prediction is no better than a random guess. Log-rank p-value is based on the log-rank test [34,35] to evaluate the null hypothesis that no difference in survival exists between the high-risk and low-risk groups. Log-rank p-value less than 0.05 means that there is significant difference between these two groups. Users have the option to split the data by N folds cross-validation, where the model is trained on the N-1 fold data and evaluated on the rest 1 fold data.

Pathway level analysis

The selected pathway features from classification or prognosis prediction, can be visualized with the *Pathview* r package [36]. Currently, any KEGG pathway can be used as the input to render pathway graphs. The top pathways are selected with the *featureSelection()* function in *Lilikoi*. Additionally, if there are corresponding gene expression profiles, they can be integrated with metabolites in *Pathview.*

The relationship between pathway and the metabolites in that particular pathway can be analyzed by single variate regression. The metabolites that are significantly associated with the pathway are displayed as bar graphs and top tables. All pathway features and their significantly associated metabolites are visualized by a bipartite graph with Cytoscape style. Cytoscape modules are imported in *Lilikoi* by the *RCy3* R package [37].

Availability of source code and requirements

Project name: Lilikoi

Project home page: https://github.com/lanagarmire/lilikoi2

Operating system(s): Windows and macOS

Programming language: e.g., R

Other requirements: e.g., R ≥ 3.5.0

Dependencies: car, caret, dplyr, gbm, ggplot2, glmnet, h2o, impute, infotheo, limma, M3C, Metrics, MLmetrics, parallel, pathifier, pathview, plyr, preprocessCore, pROC, RCy3, reticulate, reshape, RWeka, scales, stringr, survminer, survival

License: GPL-2

Lilikoi v2.0 source code with documentation and scripts to run testing data are available at https://github.com/lanagarmire/lilikoi2. Lilikoi v2.0 R package is submitted to CRAN team and upon passing, it will be expected to be available at: https://cran.r-project.org/web/packages/lilikoi/index.html.

RESULTS

Overview of updated functionalities in *Lilikoi v2.0*

Lilikoi v2.0 package is a significant upgrade of the previous version. It keeps all four modules in the original *Lilikoi v1* package: feature mapper, dimension transformer, feature selector, and classification predictor [2]. However, given the recent applications of deep-learning in the metabolomics and other genomics fields [3–8], it is important to enable metabolomics researchers to investigate such new approaches. We thus implemented deep-learning as a new method in the classification module. Moreover, metabolomics have the potential to be prognosis markers [10], however, currently rarely metabolomics data analysis workflow is available to handle this issue. We herein implemented multiple methods for prognosis prediction, including *Cox-Proportional Hazard* (or *Cox-PH*) model and *Cox-nnet*, a neural-network based model [5]. Additionally, we augmented the pathway-based metabolomics analysis with metabolite-pathway relationship analysis and pathway visualization. Last but not least, we also include additional preprocessing methods for metabolomics data analysis (eg. normalization, imputation) and tools for exploratory data analysis (eg. PCA and t-SNE analysis, and source of variation analysis).

Importantly, *Lilikoi v2.0* has added the following new functionalities (marked in red text boxes), as shown in Fig. 1. A pre-processing module is added for the initial steps, where normalization and imputation are considered. A new exploratory data analysis module is also added, to enable dimension reduction analysis (PCA or t-SNE) and source of variation analysis (SOV). The classification module is amended with the new deep-learning method, along with the previously implemented machine-learning methods. Additionally, a new prognosis module is introduced in this version, where cox-PH method and a new neural-network based Cox-nnet method are implemented. Downstream analysis and interpretation of pathways is also a new add-on feature, where visualization and metabolite-pathway regression are available.

Data preprocessing and exploratory analysis

For data preprocessing, we added normalization and imputation methods. Three normalization methods (standard normalization, quantile normalization and median-fold normalization) are implemented, with median-fold normalization as the default method. For imputation of missing values, k-nearest neighbor method is the default method.

Un-supervised exploratory analysis is an important step to better understand the pattern in metabolomics data, as well as the metabolomics-phenotype relationship. To enable this, *Lilikoi* v2.0 added Principle Component Analysis (PCA) and t-Distributed Stochastic Neighbor Embedding (t-SNE) plot that help users to visualize high-dimensional metabolomics data. PCA reduces the dataset dimensions by finding out the linearly independent dimensions based on the eigenvalues and eigenvectors of the covariance matrix to represent the data. Different from the linear dimension reduction of PCA, t-SNE maps the high-dimensional data onto a low-dimensional space via a non-linear algorithm.

To investigate the metabolomics-phenotype data relationship, *Lilikoi* v2.0 has added the source of variation analysis between confounders and metabolomics data, based on ANOVA tests [18]. Any clinical confounder with F-score bigger than the error term, whose F-score is 1, needs to be adjusted for in differential metabolite tests, when using other clinical variable(s) for grouping.

Deep learning enabled classification module

Deep-learning enabled classification module is one of the highlighted functionalities of *Lilikoi v2.0*. The deep learning framework uses the same dataset and adopts the same architecture as previously described [9]. The objective is to classify the 204 ER+ samples from the 67 ER- samples. We split the data roughly 4:1 ratio into training and testing data, with 10 fold cross-validation in the training data. We repeated this process 10 times randomly, to obtain averaged metrics.

We used the metabolite features as the inputs for deep-learning based classification, along with other popular methods: LDA, SVM, RF, RPART, LOG, and GBM (Methods). As shown in Fig. 2A and Table 1, deep learning on average performs the best overall in the training data, with the significantly higher F-1 statistic value (0.95) and sensitivity (0.98) than all other methods. F-1 statistic is a good unbiased metric given the unbalanced samples in ER+ and ER- classes. However, the specificity (0.75) in the training dataset is second to the lowest (SPEC of LDA=0.72). The advantage of deep learning is more pronounced in the testing dataset (Fig. 2B and Table 1), where it achieves the highest values in AUC=0.91, SEN=0.95, and F1 statistics=0.93. Again the specificity is lower than other methods (0.69), probably due to the size of the samples. As a word of caution, the computation time to run the deep-learning method is significantly longer than other machine learning methods, and it is only beneficial when the sample size is median (in the order of hundreds).

Prognosis prediction

Deep-learning enabled prognosis prediction is another unique functionalities of *Lilikoi v2.0*, compared to other metabolomics analysis packages and toolkits. To demonstrate prognosis analysis, we used the NCI dataset as described in Methods. As the unique feature of Lilikoi is pathway-level modeling, the metabolites intensity data are first transformed to pathway level data matrix (see Methods). Penalized survival analysis using *Cox-PH* model and *Cox-nnet* were conducted. For *Cox-PH* regression, L2 norm (Ridge) penalization was applied to select featured pathways. After fitting, the prognosis index (PI) was used to separate the patient into the high-risk vs low-risk groups using the first quantile of PI as the

threshold. As shown by the Kaplan-Meier curves in Fig. 3, the *Cox-PH* model yields a C-index value of 0.65 and log rank p-value of 0.04 (Fig. 3A); *Cox-nnet* model yields slightly better results, with a C-index value of 0.66 and log rank p-value of 0.02 (Fig. 3B).

Pathway downstream analysis

We used the metabolites expression information in the aforementioned workbench breast cancer dataset PR000284 as the cpd.data input of the *pathview* function. According to our *featureSelection* results, alanine aspartate and glutamate metabolism is one of the top pathways for metabolite data. Therefore, we demonstrate the pathway visualization, based on the *Pathview* R package. using "alanine aspartate and glutamate metabolism pathway" (Fig. 4). As shown in Fig. 4, 6 metabolites in this pathway have intensities. Asparagine has increased levels in ER- patients, due to the conversion from its substrate aspartate, which is reduced in ER- patients. The reduction of aspartate in ER- patients is consistent with previous observation [38].

It is important to link the significant metabolites that contribute to the pathway features. For this, single-variate regressions between metabolites and pathways are conducted, with the workbench dataset with 207 plasma samples (126 breast cancer cases and 81 control cases). The regression results (Fig. 5) can be visualized by the partite graph, where the yellow nodes represent pathway features, and the green nodes are metabolites significantly ($p<0.05$) associated with the pathways. show how each metabolite contributes to the selected pathways. The generic term "metabolic pathways", is associated with the largest number (86) of metabolites. Among them, isopentenyl pyrophosphate has the most weight on the edge. Many pathways related to amino acid synthesis and metabolism are highlighted. Users can also elect to exam the metabolites within a particular pathway, by individual bar graphs. As an example, we show the metabolites that are associated with "alanine aspartate and glutamate metabolism pathway" (Fig. 5, insert). Citric acid, pyruvate, 5-phosphoribosylamine, glutamine, oxaloacetate and asparagine all significantly ($p<0.05$) increase in ER- patients, with coefficients of 0.043, 0.046, 0.049, 0.378, 0.575 and 0.997 from single-variate linear regressions; on the other hand, succinate and aspartate have

opposite significant decreases, with coefficients of -0.435 and -0.269. Additional bar graphs showing relationships of metabolites and all top 10 pathways are in Supplementary Figure S1.

DISCUSSION AND CONCLUSIONS

Here we report the upgrade of *Lilikoi* v2.0, a new deep-learning enabled, personalized pathway-based package for diagnosis and prognosis predictions using metabolomics data. The new version of *Lilikoi* added many new modules, ranging from data preprocessing, exploratory analysis, deep learning, prognosis prediction, and visualization. Building upon the previous work on pathway-based modeling and prediction, *Lilikoi* v2.0 allows much better exploration of pathway-based analysis using various modern analytics methods for classification and survival analysis, including deep learning implementation. Such endeavor sets Lilikoi v2.0 apart from other more conventional metabolomics analysis packages [39–41]. One of the closest comprehensive packages MetaboAnalystR [42]. Some functions are similar between the two tools, such as classification using caret packages. However, there are some very significant differences between the two, such as the functionalities mentioned above. On the other hand, MetaboAnalystR provides other functionalities, such as time-series analysis, power analysis, and network explorer, which *Lilikoi* does not have yet.

Some practical challenges still exist, leaving room for the future development of *Lilikoi*. For example, mapping rate of metabolites and pathways can be further improved, by using better matching algorithms. Also, the current best classification model in *Lilikoi* is determined by users. We would like to automatically recommend the best classification model for users. This will be dependent on training a large set of metabolomics datasets for benchmarking, beyond of the scope of this report. Despite this, we recommend the users to pay more attention to the machine learning methods that are less prone to overfitting, such as random forest, given the fact that the majority of the datasets have median sample size (in the order of hundreds). The comparison between deep-larning and other machine-learning methods shows the advantages of gained accuracy of deep-learnign metod. However, such benefit is achieved at the cost of computation time. Moreover, the higher performance of deep-learning method

is conditional on the sample sizes. Deep-learning method is superior when the patient size is at least a few hundred. Moreover, given the limited size of annotated metabolites (in the order of hundreds) in the assays, the pathway visualization is sparse, based on metabolites alone. Integration between metabolomics and other genomics data types is helpful to fill in the missing information, especially with the aid of deep-learning and machine-learning ensemble tools, such as DeepProg models that are developed by us and others [3,4,8,43].

ACKNOWLEDGEMENTS

We thank Dr. Fadhl Alakwaa for providing help and valuable discussions in developing version 2 of this package. This research was supported by grants K01ES025434 awarded by NIEHS through funds provided by the trans-NIH Big Data to Knowledge (BD2K) initiative (www.bd2k.nih.gov), R01 LM012373 and R01 LM012907 awarded by NLM, and R01 HD084633 awarded by NICHD to L.X. Garmire.

REFERENCES

1. Spicer R, Salek RM, Moreno P, Cañueto D, Steinbeck C. Navigating freely-available software tools for metabolomics analysis. Metabolomics. 2017;13:106.

2. Al-Akwaa FM, Yunits B, Huang S, Alhajaji H, Garmire LX. Lilikoi: an R package for personalized pathway-based classification modeling using metabolomics data. Gigascience [Internet]. 2018;7. Available from: http://dx.doi.org/10.1093/gigascience/giy136

3. Chaudhary K, Poirion OB, Lu L, Garmire LX. Deep Learning-Based Multi-Omics Integration Robustly Predicts Survival in Liver Cancer. Clin Cancer Res. 2018;24:1248–59.

4. Poirion OB, Chaudhary K, Garmire LX. Deep Learning data integration for better risk stratification models of bladder cancer. AMIA Jt Summits Transl Sci Proc. 2018;2017:197–206.

5. Ching T, Zhu X, Garmire LX. Cox-nnet: An artificial neural network method for prognosis prediction of high-throughput omics data. PLoS Comput Biol. 2018;14:e1006076.

6. Arisdakessian C, Poirion O, Yunits B, Zhu X, Garmire LX. DeepImpute: an accurate, fast, and scalable deep neural network method to impute single-cell RNA-seq data. Genome Biol. 2019;20:211.

7. Chen B, Garmire L, Calvisi DF, Chua M-S, Kelley RK, Chen X. Harnessing big “omics” data and AI for drug discovery in hepatocellular carcinoma. Nat Rev Gastroenterol Hepatol. 2020;17:238–51.

8. Poirion OB, Chaudhary K, Huang S, Garmire LX. Multi-omics-based pan-cancer prognosis prediction using an ensemble of deep-learning and machine-learning models [Internet]. Available from: http://dx.doi.org/10.1101/19010082

9. Alakwaa FM, Chaudhary K, Garmire LX. Deep Learning Accurately Predicts Estrogen Receptor Status in Breast Cancer Metabolomics Data. J Proteome Res. 2018;17:337–47.

10. Terunuma A, Putluri N, Mishra P, Mathé EA, Dorsey TH, Yi M, et al. MYC-driven accumulation of 2-hydroxyglutarate is associated with breast cancer prognosis. J Clin Invest. 2014;124:398–412.

11. Xie G. Breast Cancer GC/MS and LC/MS plasma data from City of Hope Hospital [Internet]. 2016 [cited 2020 Apr 14]. Available from: https://www.metabolomicsworkbench.org

12. Budczies J, Denkert C, Müller BM, Brockmöller SF, Klauschen F, Györffy B, et al. Remodeling of central metabolism in invasive breast cancer compared to normal breast tissue - a GC-TOFMS based metabolomics study. BMC Genomics. 2012;13:334.

13. Bolstad B. preprocessCore: A collection of pre-processing functions. R package version 1.48.0 [Internet]. 2019. Available from: https://github.com/bmbolstad/preprocessCore

14. Hastie T, Tibshirani R, Narasimhan B, Chu G. impute: Imputation for microarray data [Internet]. 2019 [cited 2020 Apr 14]. Available from: http://bioconductor.org/packages/impute/

15. Pearson K. LIII. On lines and planes of closest fit to systems of points in space. The London, Edinburgh, and Dublin Philosophical Magazine and Journal of Science. 1901;2:559–72.

16. van der Maaten L, Hinton G. Visualizing Data using t-SNE. Journal of Machine Learning Research. 2008;9:2579–605.

17. John CR, Watson D, Russ D, Goldmann K, Ehrenstein M, Pitzalis C, et al. M3C: Monte Carlo reference-based consensus clustering. Sci Rep. 2020;10:1816.

18. Fox J, Weisberg S. An R Companion to Applied Regression. SAGE Publications; 2018.

19. Ching T, Ha J, Song M-A, Tiirikainen M, Molnar J, Berry MJ, et al. Genome-scale hypomethylation in the cord blood DNAs associated with early onset preeclampsia. Clin Epigenetics. 2015;7:21.

20. Ching T, Song M-A, Tiirikainen M, Molnar J, Berry M, Towner D, et al. Genome-wide hypermethylation coupled with promoter hypomethylation in the chorioamniotic membranes of early onset pre-eclampsia. Mol Hum Reprod. 2014;20:885–904.

21. Marco-Ramell A, Palau-Rodriguez M, Alay A, Tulipani S, Urpi-Sarda M, Sanchez-Pla A, et al. Evaluation and comparison of bioinformatic tools for the enrichment analysis of metabolomics data. BMC Bioinformatics [Internet]. BioMed Central; 2018 [cited 2020 Nov 17];19. Available from: https://www.ncbi.nlm.nih.gov/pmc/articles/PMC5749025/

22. Drier Y, Sheffer M, Domany E. Pathway-based personalized analysis of cancer. Proc Natl Acad Sci U S A. 2013;110:6388–93.

23. Hastie T, Stuetzle W. Principal Curves. J Am Stat Assoc. Taylor & Francis; 1989;84:502–16.

24. Huang S, Chong N, Lewis NE, Jia W, Xie G, Garmire LX. Novel personalized pathway-based metabolomics models reveal key metabolic pathways for breast cancer diagnosis. Genome Med. 2016;8:34.

25. Huang S, Yee C, Ching T, Yu H, Garmire LX. A novel model to combine clinical and pathway-

based transcriptomic information for the prognosis prediction of breast cancer. PLoS Comput Biol. 2014;10:e1003851.

26. The H2O. ai Team. h2o: R Interface for H2O. R package version 3.1.0.99999 [Internet]. 2015. Available from: http://www.h2o.ai

27. The H2O. ai Team. h2o: h2o: Python Interface for H2O. Python package version 3.1.0.99999 [Internet]. 2015. Available from: http://www.h2o.ai

28. The H2O. ai Team. H2O: Scalable Machine Learning. Version 3.1.0.99999 [Internet]. 2015. Available from: http://www.h2o.ai

29. Cox DR. Regression Models and Life-Tables [Internet]. Journal of the Royal Statistical Society. Series B (Methodological). 1992. p. 527–41. Available from: https://www.jstor.org/stable/2985181

30. Friedman J, Hastie T, Tibshirani R. Regularization Paths for Generalized Linear Models via Coordinate Descent [Internet]. Journal of Statistical Software. 2010. p. 1–22. Available from: http://www.jstatsoft.org/v33/i01/

31. JJ Allaire, Kevin Ushey, Yuan Tang, and Dirk Eddelbuettel. reticulate: R Interface to Python [Internet]. 2017. Available from: https://github.com/rstudio/reticulate

32. Alboukadel Kassambara, Marcin Kosinski, Przemyslaw Biecek, Scheipl Fabian. Drawing Survival Curves using "ggplot2". R package survminer version 0.4.6 [Internet]. Comprehensive R Archive Network (CRAN); 2019. Available from: https://CRAN.R-project.org/package=survminer

33. Harrell FE. Evaluating the Yield of Medical Tests [Internet]. JAMA: The Journal of the American Medical Association. 1982. p. 2543. Available from: http://dx.doi.org/10.1001/jama.1982.03320430047030

34. Mantel N. Evaluation of survival data and two new rank order statistics arising in its consideration. Cancer Chemother Rep. 1966;50:163–70.

35. Peto R, Peto J. Asymptotically Efficient Rank Invariant Test Procedures [Internet]. Journal of the Royal Statistical Society. Series A (General). 1972. p. 185. Available from: http://dx.doi.org/10.2307/2344317

36. Luo W, Brouwer C. Pathview: an R/Bioconductor package for pathway-based data integration and visualization [Internet]. Bioinformatics. 2013. p. 1830–1. Available from: http://dx.doi.org/10.1093/bioinformatics/btt285

37. Gustavsen JA, Pai S, Isserlin R, Demchak B, Pico AR. RCy3: Network biology using Cytoscape from within R. F1000Res. 2019;8:1774.

38. Fan Y, Zhou X, Xia T-S, Chen Z, Li J, Liu Q, et al. Human plasma metabolomics for identifying differential metabolites and predicting molecular subtypes of breast cancer. Oncotarget. 2016;7:9925–38.

39. Liebal UW, Phan ANT, Sudhakar M, Raman K, Blank LM. Machine Learning Applications for Mass Spectrometry-Based Metabolomics. Metabolites [Internet]. 2020;10. Available from: http://dx.doi.org/10.3390/metabo10060243

40. O'Shea K, Misra BB. Software tools, databases and resources in metabolomics: updates from 2018 to 2019. Metabolomics. 2020;16:36.

41. Ghosh T, Zhang W, Ghosh D, Kechris K. Predictive Modeling for Metabolomics Data. Methods Mol Biol. 2020;2104:313–36.

42. Chong J, Xia J. MetaboAnalystR: an R package for flexible and reproducible analysis of metabolomics data. Bioinformatics. 2018;34:4313–4.

43. Huang S, Chaudhary K, Garmire LX. More Is Better: Recent Progress in Multi-Omics Data Integration Methods. Front Genet. 2017;8:84.

FIGURES AND LEGENDS

Figure 1: The workflow of *Lilikoi v2.0* package. *Lilikoi v2.0* is composed of seven modules: feature mapper, pre-processing, dimension transformer, exploratory analysis, classification, prognosis model, and pathway analysis. Input data requires metabolomics data matrix, and one column of a categorical variable to specify case/control status for each subject (for classification), or survival information (for prognosis analysis). Feature mapping converts metabolite names to standardized metabolic IDs (e.g. HMDB IDs) and then transforms them into pathway names. Pre-processing enables three normalization methods (standard normalization, quantile normalization and median-fold normalization) and one knn imputation method. The pink boxes are new functionalities added to *Lilikoi v2.0*. Blue boxes are pre-existing modules in *Lilikoi v1*. Dashed box indicates an optional step.

Figure 2: Model evaluation on deep learning (DL) and other machine learning techniques. (A) metrics on training data sets; (B) metrics on testing data sets. Abbreviations: deeplearning (DL), generalized boosted model (GBM), linear discriminant analysis (LDA), logistic regression (LOG), random forest (RF), recursive partitioning and regression analysis (RPART), support vector machine (SVM). *: p-value < 0.05 (one-tail t-test) compared to the same metric in DL; **:p-value < 0.01 (one-tail t-test); AUC, or accuracy, measures how the model can distinguish between classes. SEN, or Sensitivity, measures the capability of a model to correctly identify cases or diseases. SPEC, or Specificity, measures the capability of a model to correctly identify controls or normal status. F1-statistics measures the accuracy of a model. Balanced accuracy is the mean of specificity and sensitivity, a good metric to consider when the sample sizes in cases and controls are not balanced.

Figure 3: Comparison of Kaplan-Meier curves resulting from Cox-PH and Cox-nnet. The samples are dichotomized into 2 risk groups by the first quantile of the prognosis index (PI) score. (A) Cox-PH

model. (B) Cox-nnet model with 3-layer neural network: one input layer, one fully connected hidden layer and the output layer.

Figure 4: Pathway visualization: alanine aspartate and glutamate metabolism pathway. Color scheme is based on log2 transformed ratio of the mean values of ER- samples over ER+ samples. The pathway rendering is done by the *Pathview* R package.

Figure 5: Metabolite-pathway relationship analysis. (A) Bipartite plot with top 10 pathways and corresponding metabolites. Cyan and yellow nodes indicate metabolites and pathways, respectively. Red and blue edges are negative (-) and positive (+) associations, respectively. Thicker edges indicate higher levels of association. (B) Bar plots of the relationship between the Alanine, Aspartate And Glutamate Metabolism pathway and its corresponding metabolites. *: p<0.05; **: p<0.01; ***:p<0.001.

TABLES

Table 1. Performance of classification models on training and hold-out testing dataset

Data Set	Algorithm	AUC	SENS	SPEC	F1-Statistic	Balanced accuracy	Computing time/run (sec)
Training	DL	**0.909**	**0.978**	0.747	**0.952**	0.777	570.68
	GBM	0.906	0.600	0.945	0.666	0.772	8.291
	LDA	**0.700**	0.583	0.718	0.478	0.651	3.118
	LOG	0.906	0.608	0.946	0.681	0.777	5.394
	RF	0.892	0.568	0.946	0.648	0.757	21.340
	RPART	0.801	0.605	0.895	0.620	0.750	3.525
	SVM	0.905	0.663	0.920	0.688	0.791	4.941

Testing	DL	**0.912**	**0.954**	0.688	**0.930**	0.747	1.844
	GBM	0.878	0.560	0.939	0.639	0.749	0.0152
	LDA	0.745	0.627	0.754	0.527	0.691	0.0149
	LOG	0.873	0.550	0.943	0.634	0.747	0.0184
	RF	0.870	0.578	0.938	0.643	0.758	0.0181
	RPART	0.767	0.609	0.861	0.589	0.735	0.0257
	SVM	0.883	0.653	0.927	0.693	0.790	0.0218

Figure1

Click here to access/download;Figure;Lilikoi Figure 1.docx

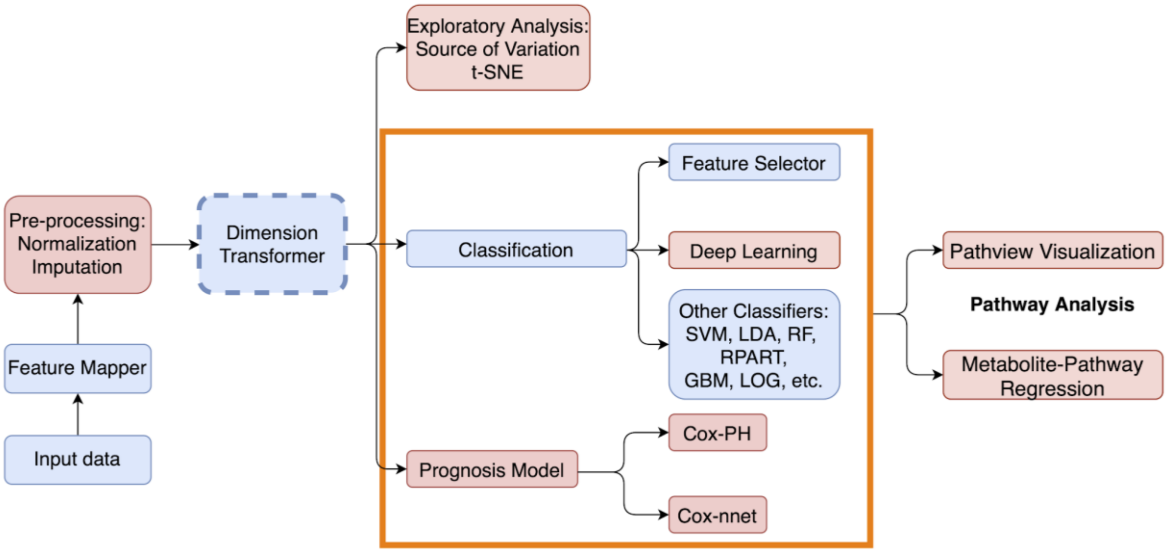

Click here to access/download;Figure;Lilikoi Figure 2.docx

Fig2 A

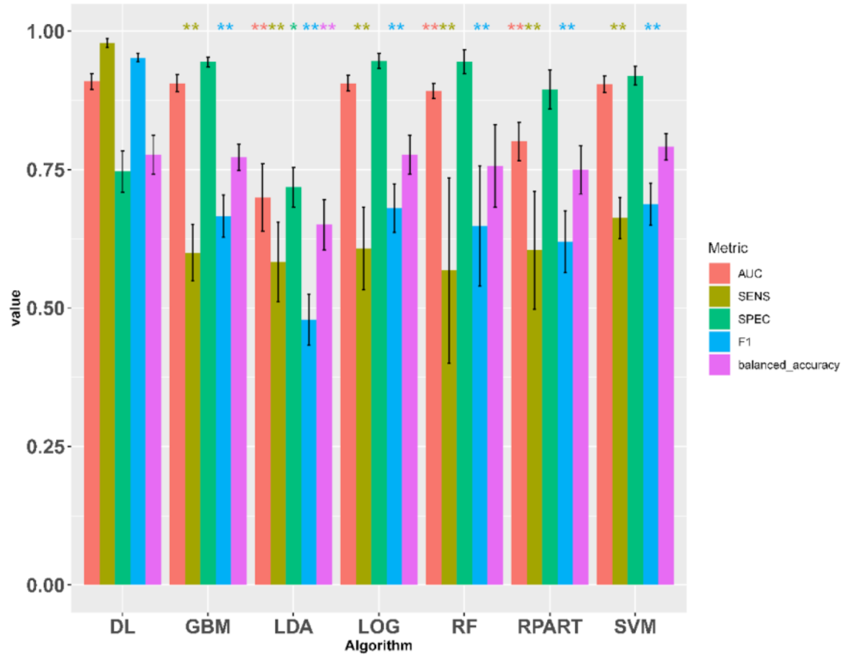

B

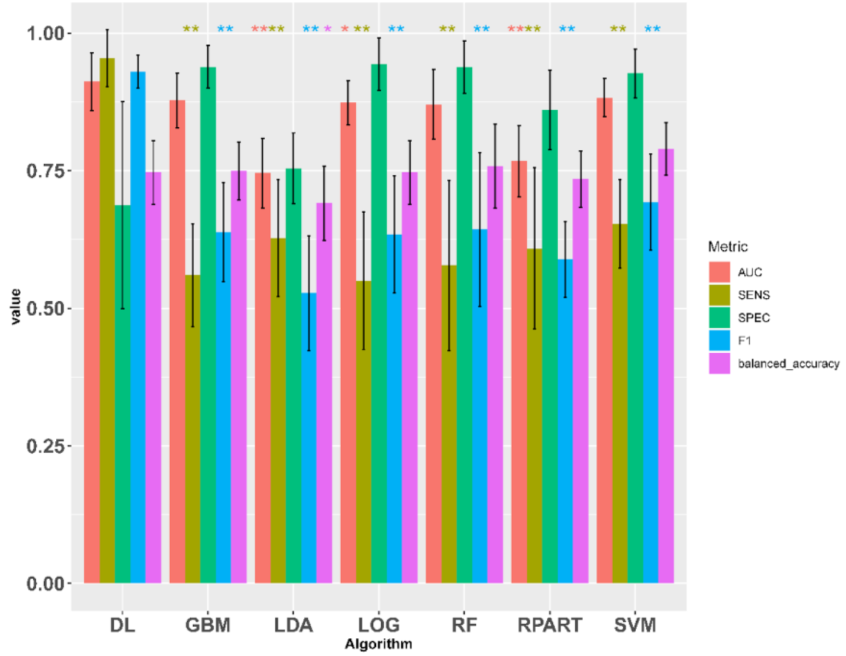

Figure 3 Click here to access/download;Figure;Lilikoi Figure 3.docx

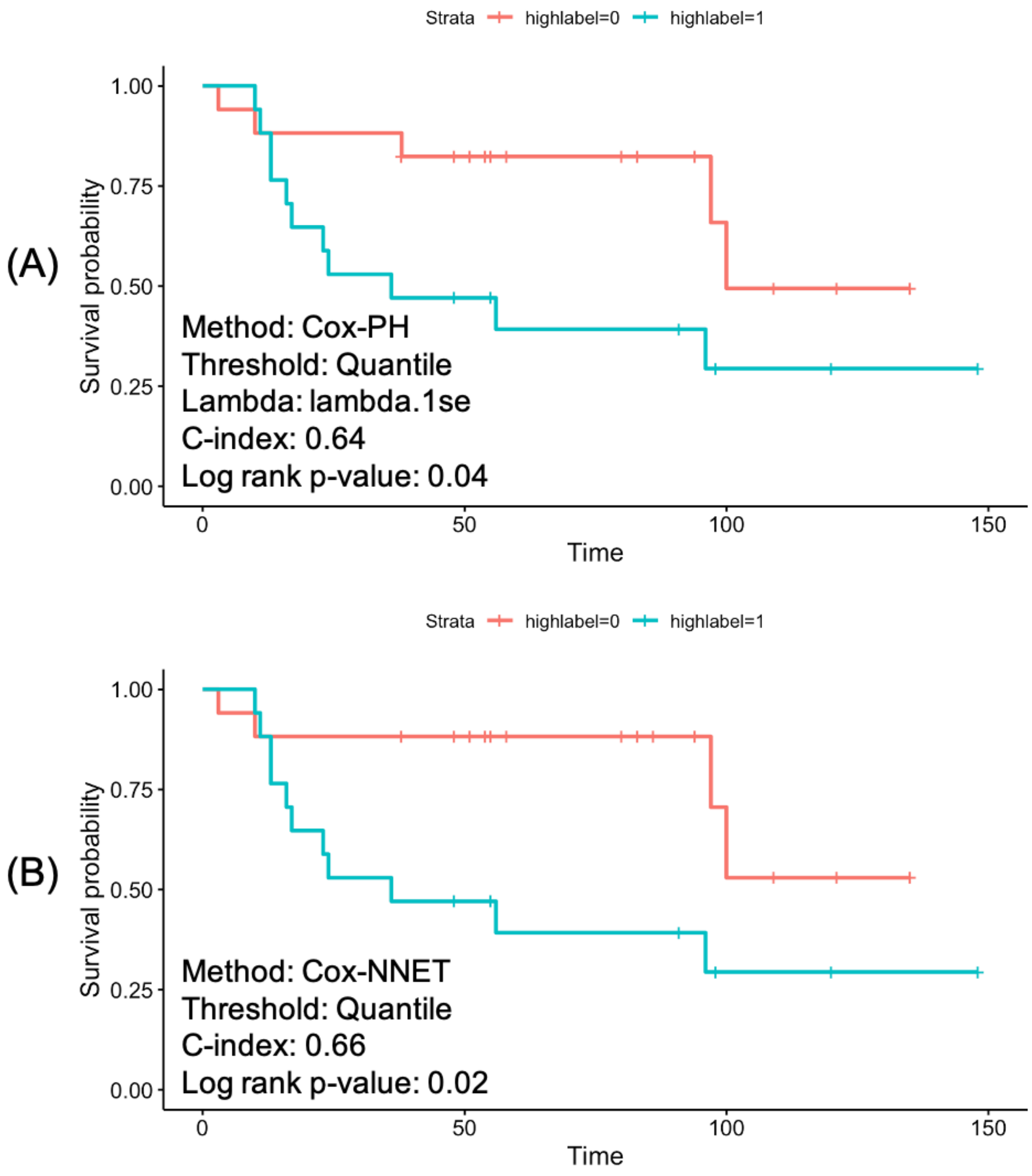

Figure 4

Click here to access/download;Figure;Lilikoi Figure 4.docx

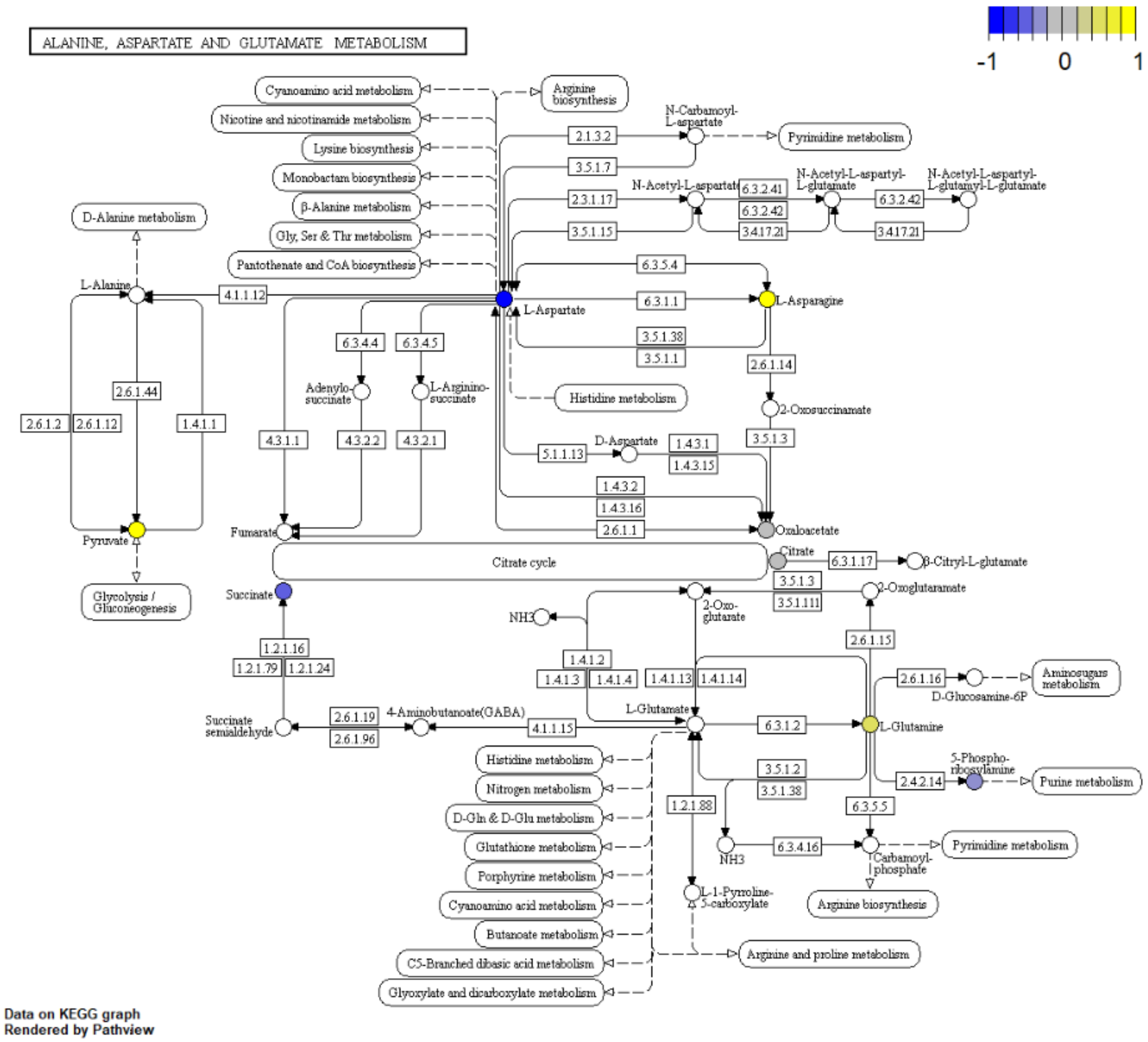

Figure 5 Click here to access/download;Figure;Lilikoi Figure 5.docx

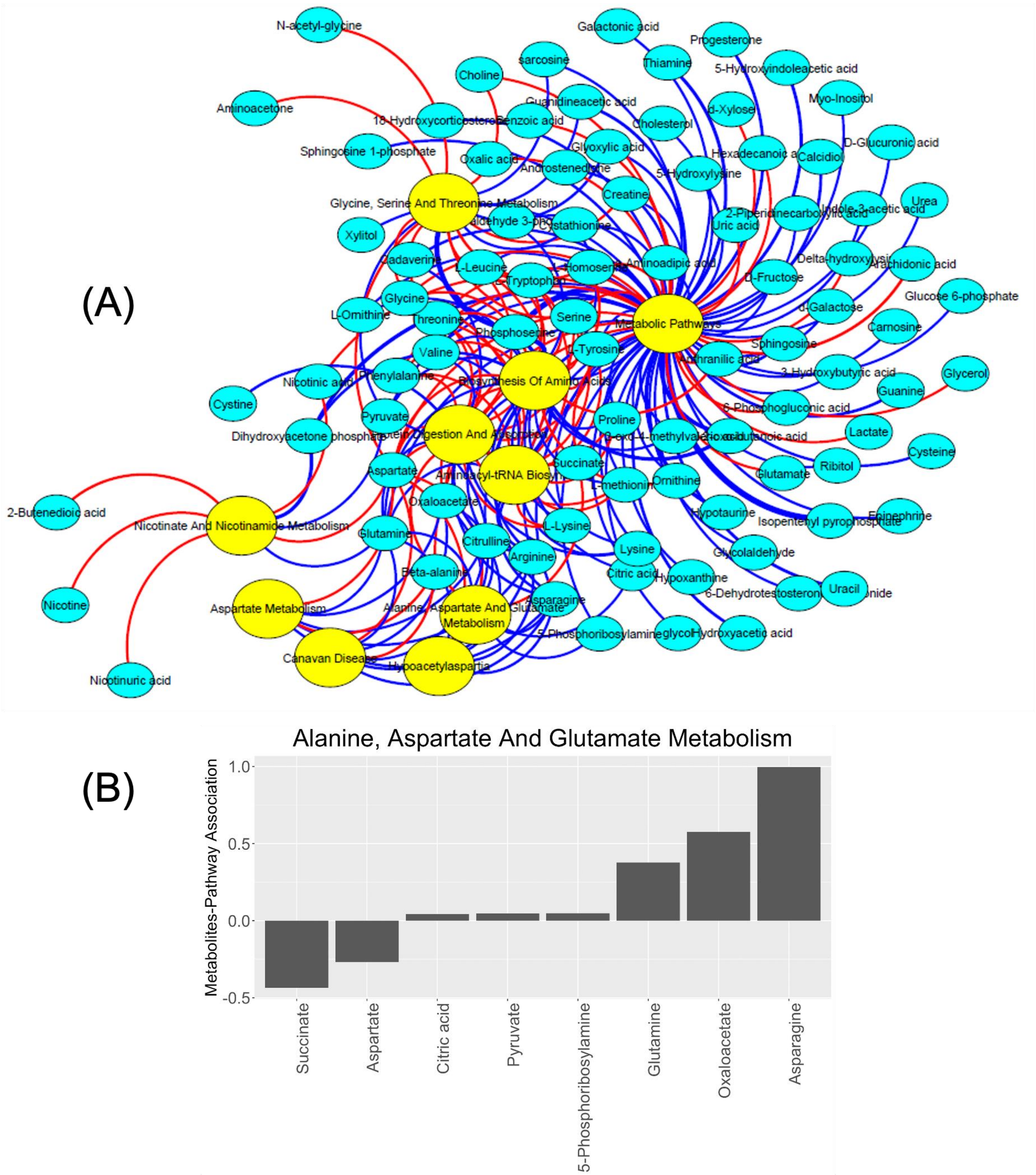

Click here to access/download
Supplementary Material
Lilikoi Supplementary.docx

Click here to access/download

Supplementary Material

Lilikoi2_Supplementary_er162_Identifiers.csv

Supplementary Material JCI71180sd2 dataset identifiers

Click here to access/download

Supplementary Material

Lilikoi2_Supplementary_JCI71180sd2_Identifiers.csv

Supplementary Material plasma dataset identifiers

Click here to access/download
Supplementary Material
Lilikoi2_Supplementary_plasma_Identifiers.csv

Click here to access/download
Supplementary Material
Lilikoi2_supplement_Lilikoi V2.0 Manual.pdf

Personal Cover
Click here to access/download;Personal Cover;Cover_Letter_Lilikoi2_GigaScience.docx

MICHIGAN MEDICINE
UNIVERSITY OF MICHIGAN

University of Michigan Medical School
Department of Computational Medicine & Bioinformatics

10/01/2020

Dear Editor,

We would like to sincerely thank the reviewers who gave us very helpful and constructive suggestions on my initial submission of “Lilikoi V2.0: a deep-learning enabled, personalized pathway-based R package for diagnosis and prognosis predictions using metabolomics data”.

Attached please see revision where the changes are in blue font, along with the point-to-point answers (in red) to fully address the reviewers’ questions.

We hope this work is satisfactory. Please feel free to contact me: lgarmire@med.umich.edu, if you have any questions.

Sincerely,

Lana Garmire, PhD

Associate Professor

Department of Computational Medicine and Bioinformatics

Medical School, University of Michigan, Ann Arbor, 48105, USA
