# Supplementary material for: Lilikoi V2.0: a deep learning–enabled, personalized pathway-based R package for diagnosis and prognosis predictions using metabolomics data
Source: Gigascience. 2021 Jan 23;10(1):giaa162. doi: 10.1093/gigascience/giaa162 (PMC7825009; doi:10.1093/gigascience/giaa162)

**SUPPLEMENTARY FIGURE**

**Figure S1: relationships between metabolites and all ten pathways.** Pathways are selected by the featureSelection function in *lilikoi,* with selection threshold of 0.54 and decision tree method of gain ratio.


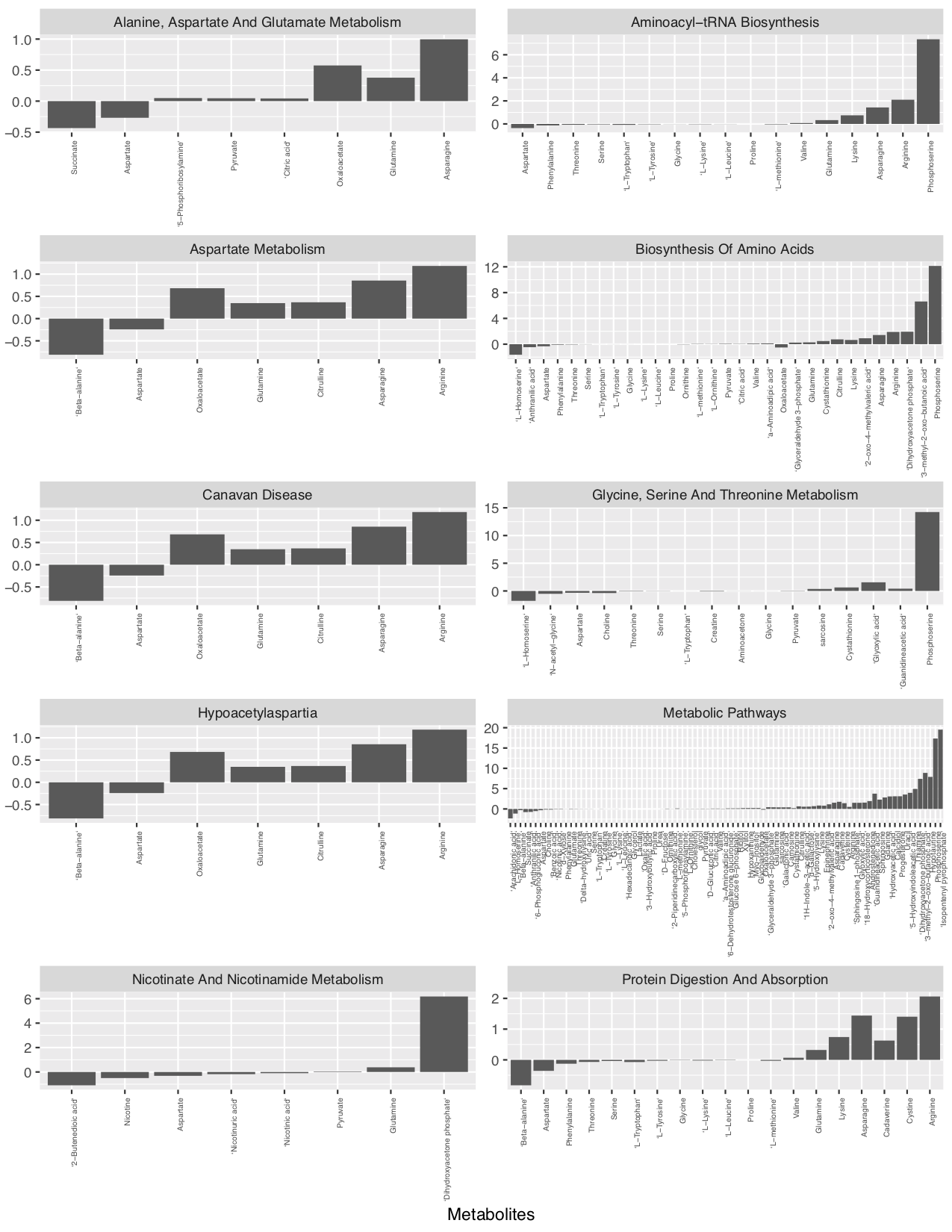

Supplement: giaa162_Supplemental_Files [file giaa162_supplemental_files.zip › Lilikoi Supplementary.docx]
