# Supplementary material for: Lilikoi V2.0: a deep learning–enabled, personalized pathway-based R package for diagnosis and prognosis predictions using metabolomics data
Source: Gigascience. 2021 Jan 23;10(1):giaa162. doi: 10.1093/gigascience/giaa162 (PMC7825009; doi:10.1093/gigascience/giaa162)
Supplement: giaa162_Supplemental_Files [file giaa162_supplemental_files.zip › Lilikoi2_supplement_Lilikoi V2.0 Manual.pdf]

Lilikoi V2.0

Xinying Fang

2020-09-30

Introduction

Previously we developed Lilikoi, a personalized pathway-based method to classify diseases using metabolomics data. Given the new trends of computation in the metabolomics field, here we report the next version of Lilikoi as a significant upgrade. The new Lilikoi v2.0 R package has implemented a deep-learning method for classification, in addition to popular machine learning methods. It also has several new modules, including the most significant addition of prognosis prediction, implemented by Cox-PH model and the deep-learning based Cox-nnet model. Additionally, Lilikoi v2.0 supports data preprocessing, exploratory analysis, pathway visualization and metabolite-pathway regression. In summary, Lilikoi v2.0 is a modern, comprehensive package to enable metabolomics analysis in R programming environment.

Installation

```
install.packages("lilikoi")
library(lilikoi)
```

Load data

The recommended format of dataset is csv. Columns are metabolites and rows are samples. Case and control labels should be saved in the second column.

The Loaddata function will load the dataset. For the convenience of further analysis, we extract the output to be a dataset called “Metadata” and a list of compound names called “dataSet”.

```
dt <- lilikoi.Loaddata(file=system.file("extdata", "plasma_breast_cancer.csv", package =
"lilikoi2"))
Metadata <- dt$Metadata
dataSet <- dt$dataSet
```

Transform the metabolite names to the HMDB ids using Lilikoi MetaTOpathway function

Lilikoi allows the user to input any kind of metabolite IDs including metabolites names (‘name’) along with synonyms, KEGG IDs (‘kegg’), HMDB IDs (‘hmdb’) and PubChem IDs (‘pubchem’). If the metabolites have a standard names as ID, Lilikoi will match these names among 100 k saved database, if there are not any hits, Lilikoi will perform fuzzy matching to find the closest matching for this metabolite.

```
convertResults=lilikoi.MetaTOpathway('name')
Metabolite_pathway_table = convertResults$table
head(Metabolite_pathway_table)
```

Select the most signficant pathway related to phenotype.

A specific pathway dysregulation score (PDS) is inferred to measure the abnormity for each sample in each pathway. For each pathway, the samples are mapped in a high dimensional principal component space and a principal curve is constructed along the samples and smoothed. The PDS score measures the distance from the projected dot along the curve to the centroid of normal samples (origin point of the curve).

```
selected_Pathways_Weka= lilikoi.featuresSelection(PDSmatrix,threshold= 0.54,method="gain")
selected_Pathways_Weka
```

Data preprocessing

Lilikoi v2.0 enables users to perform three normalization methods (standard normalization, quantile normalization and median-fold normalization) and imputation for missing metabolic expressions.

```
# Standard Normalization
lilikoi.preproc_norm(inputdata=Metadata, method="standard")
lilikoi.preproc_norm(inputdata=Metadata, method="quantile")
lilikoi.preproc_norm(inputdata=Metadata, method="median")
```

We also implemented imputation function using the K-Nearest Neighbors method.

```
# KNN Imputation
lilikoi.preproc_knn(inputdata=Metadata,method=c("knn"))
```

Exploration analysis

The exploration analysis in lilikoi v2.0 performs source of variation analysis with the metabolites data frame and demographic data frame provided by users. Users can choose to implement Principal Component Analysis(PCA) to extract the most important information in high-dimensional datasets and the t-SNE plot to visualize the metabolites data. The PCA and t-SNE plots can be enabled by setting those flags values to TRUE.

```
lilikoi.explr(data, demo.data, pca=TRUE, tsne=FALSE)
```

Machine learning

We include deep learning for classifciation in this version of lilikoi based on the h2o package.It used multi-layer Neural Network and Stochastic Gradient Descent to predict the diagnosis results. In order to reduce

redundancy in setting parameters, we only open the configuration for general machine learning setting. If users want to change the settings of the deep learning feature, they would need to change it in the source code.

```
lilikoi.machine_learning(MLmatrix = Metadata, measurementLabels = Metadata$Label,
                         significantPathways = 0,
                         trainportion = 0.8, cvnum = 10)
```

Prognosis prediction

Prognosis prediction is enabled in Lilikoi v2. It is implemented in both metabolite level and pathway level with Cox-PH model or Cox-NNET model. Users should provide vectors of survival results and survival time. “percent” automatically split the dataset to be training and testing datasets. “alpha” gives users choices for Lasso, Ridge or Elastic net penalizations. “nfold” indicates the fold number for cross validation. “cvlambda” determines the lambda for prediction, “lambda.min” or “lambda.1se”. “method” determines the prognosis index, “quantile”, “quantile” or “ratio”.

```
# Set up prognosis function arguments
# Before running Cox-nnet, users need to provide the directory for python3 and the inst file
in lilikoi
path = path.package('lilikoi', quiet = FALSE) # path = "lilikoi/inst/", use R to run
path = file.path(path, 'inst')

python.path = "/Library/Frameworks/Python.framework/Versions/3.8/bin/python3"


event = jcevent
time = jctime
percent = NULL
exprdata = exprdata_tumor
alpha = 0
nfold = 5
method = "quantile"
cvlambda = NULL
coxnet = TRUE
coxnet_method = "gradient"

library(reticulate)

lilikoi.prognosis(event, time, exprdata, percent=percent, alpha=0, nfold=5,
method="quantile",

cvlambda=cvlambda,python.path=python.path,path=path,coxnet=TRUE,coxnet_method="gradient")
```

Pathway level analysis

Pathview plot

The selected pathway features from classification pf prognosis prediction can be visualized in Lilikoi. Any KEGG pathway can be used as the inout to render pathway graphs. “metamat” is the metabolites expression data matrix. “sampleinfo” is a vector of sample group, with element names as sample IDs. “grouporder” is a vector with 2 elements of reference and experimental group names.

```
metamat <- t(t(Metadata[, -1]))
metamat <- log2(metamat)
sampleinfo <- Metadata$Label
names(sampleinfo) <- rownames(Metadata)
grouporder <- unique(Metadata$Label)

lilikoi.KEGGplot(metamat = metamat, sampleinfo = sampleinfo, grouporder = grouporder,
                 pathid = '00250', specie = 'hsa',
                 filesuffix = 'GSE16873',
                 Metabolite_pathway_table = Metabolite_pathway_table)
```

Metabolites-pathway relationship

We use single variate regression to reveal the relationship between pathways and their corresponding metabolites. All pathway features and their significantly associated metabolites are visualized by a bipartite graph with Cytoscape style.

```
lilikoi.meta_path(PDSmatrix = PDSmatrix, selected_Pathways_Weka = selected_Pathways_Weka,
Metabolite_pathway_table = Metabolite_pathway_table)
```
