# Supplementary figures and images for: Lilikoi V2.0: a deep learning–enabled, personalized pathway-based R package for diagnosis and prognosis predictions using metabolomics data
Source: Gigascience. 2021 Jan 23;10(1):giaa162. doi: 10.1093/gigascience/giaa162 (PMC7825009; doi:10.1093/gigascience/giaa162)

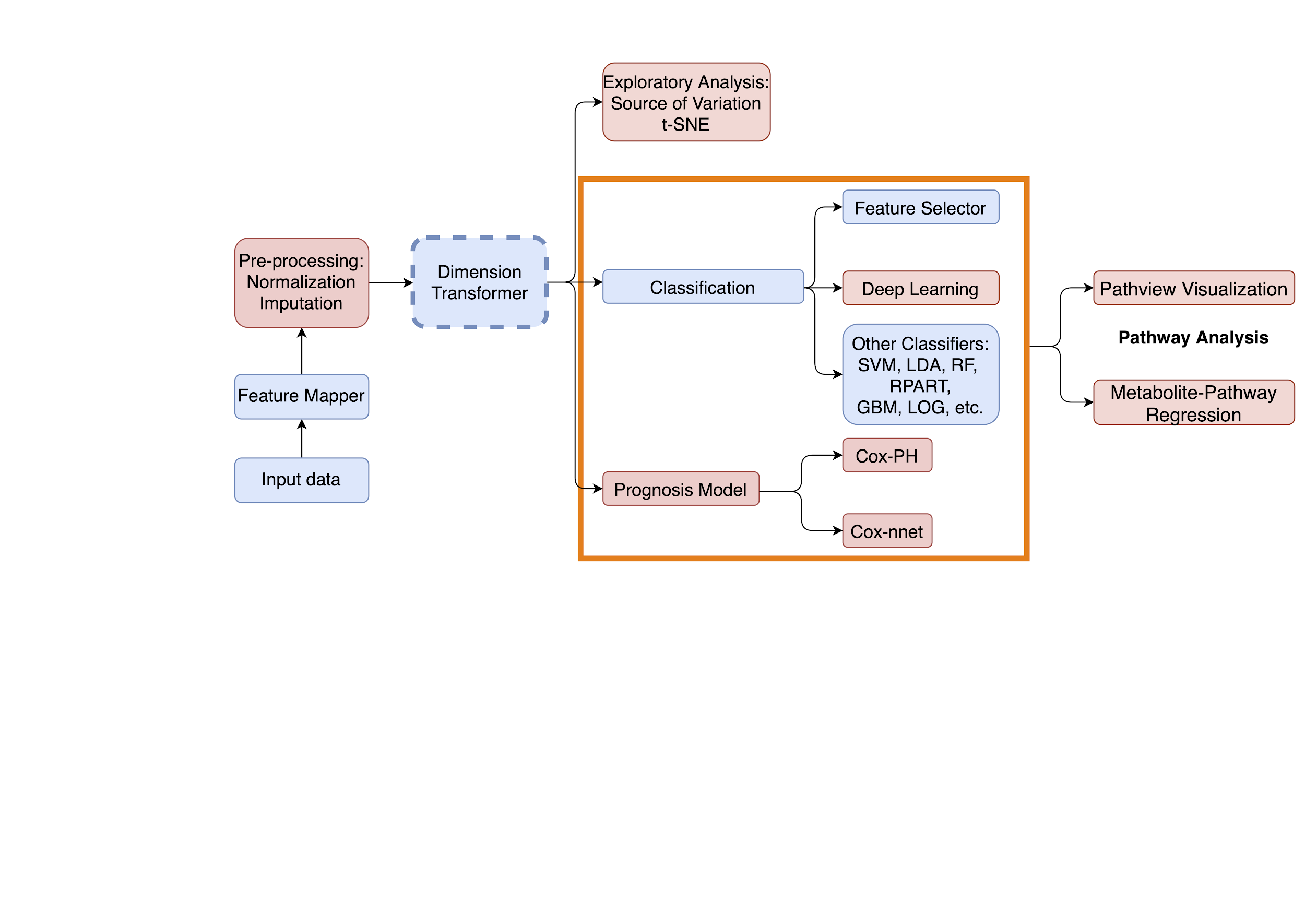

Supplement: giaa162_Supplemental_Files [file giaa162_supplemental_files.zip › Lilikoi Figure 1.docx]

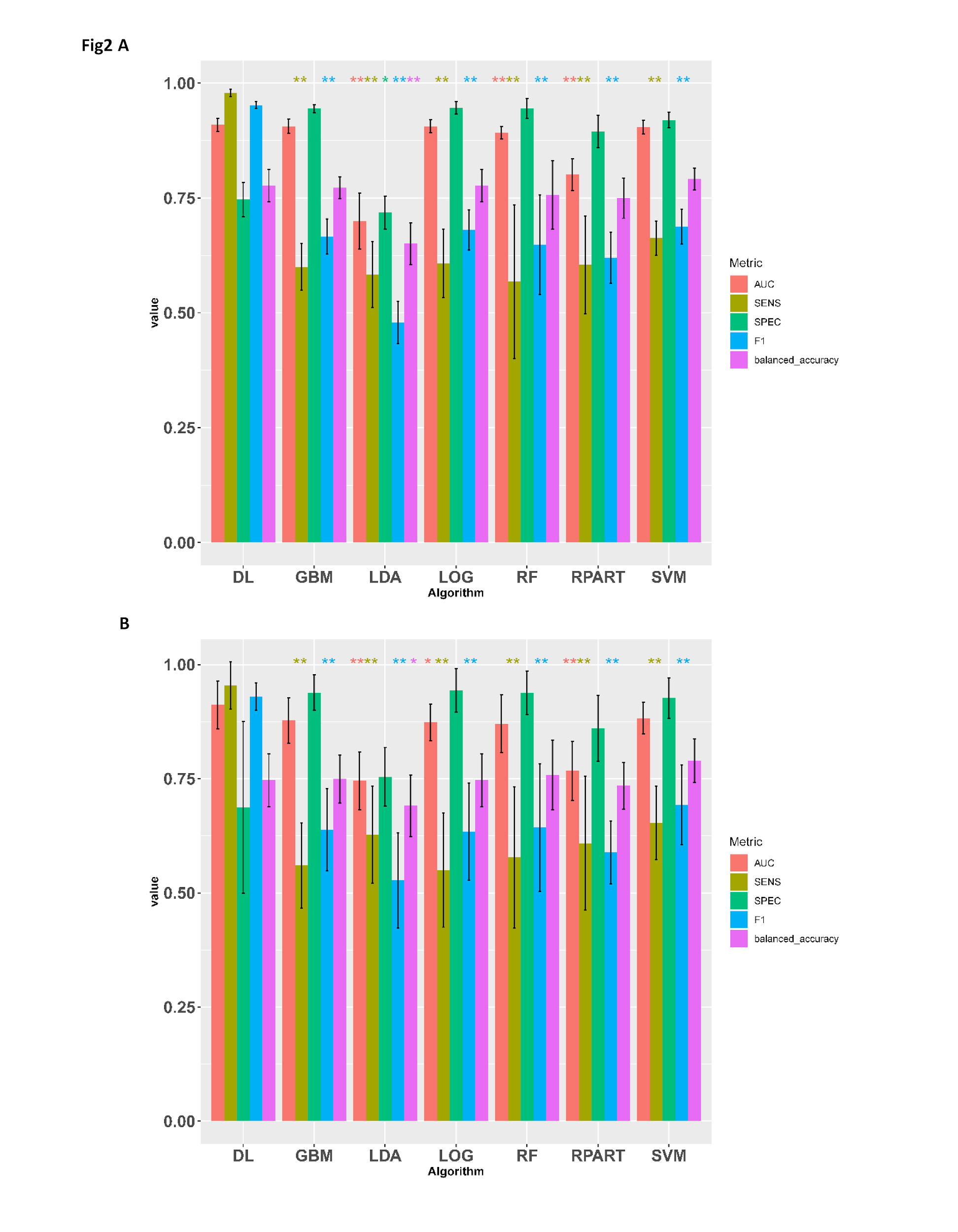

Supplement: giaa162_Supplemental_Files [file giaa162_supplemental_files.zip › Lilikoi Figure 2.docx]

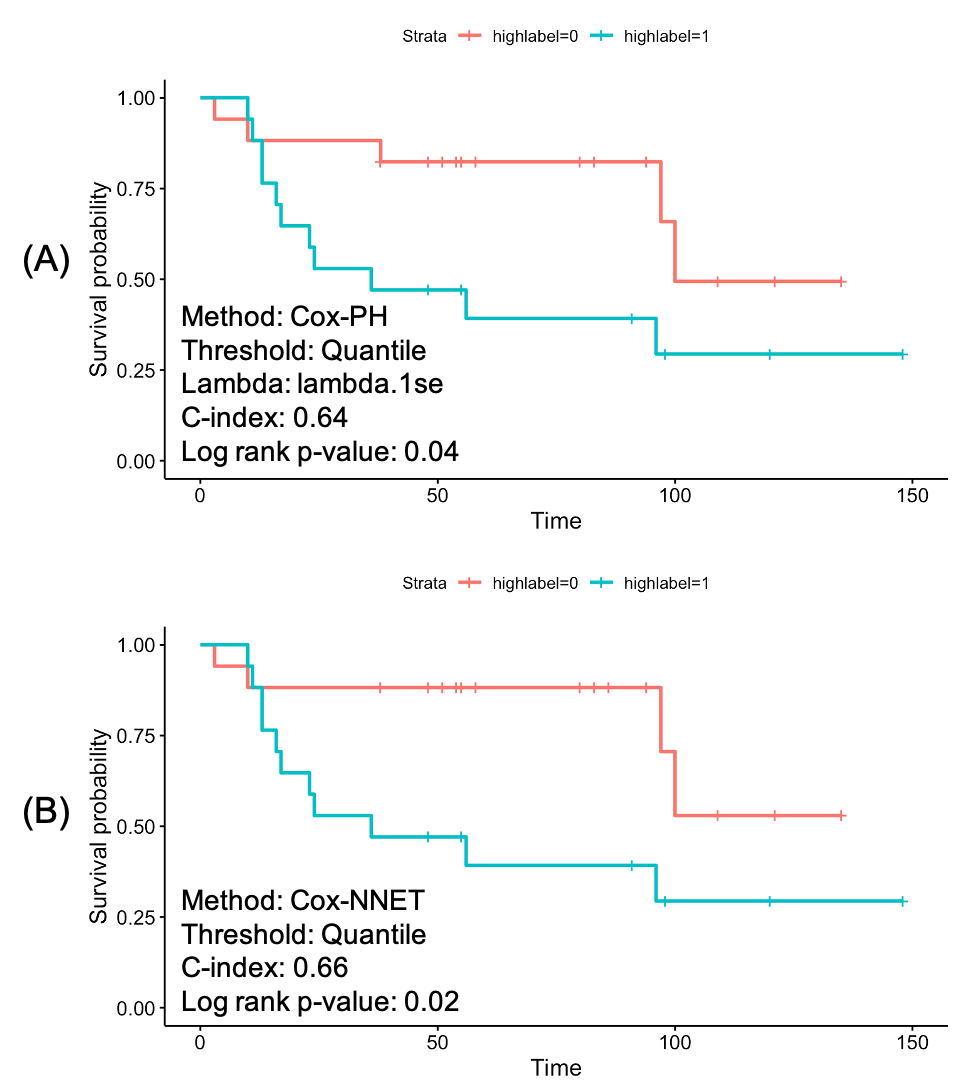

Supplement: giaa162_Supplemental_Files [file giaa162_supplemental_files.zip › Lilikoi Figure 3.docx]

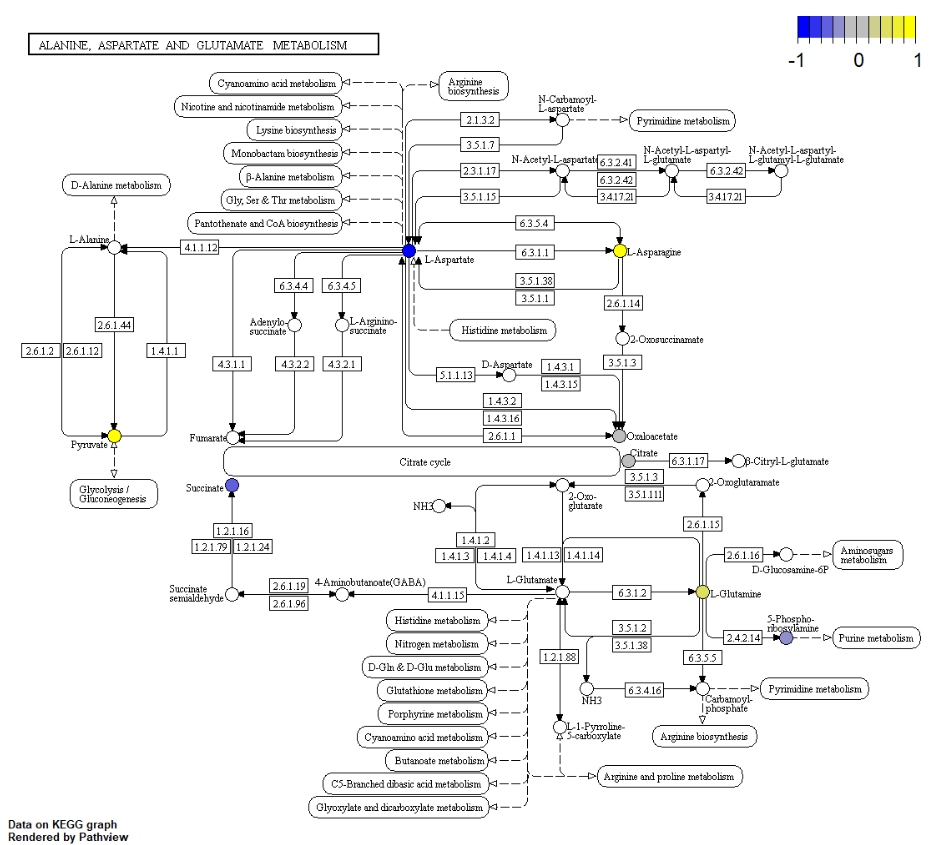

Supplement: giaa162_Supplemental_Files [file giaa162_supplemental_files.zip › Lilikoi Figure 4.docx]

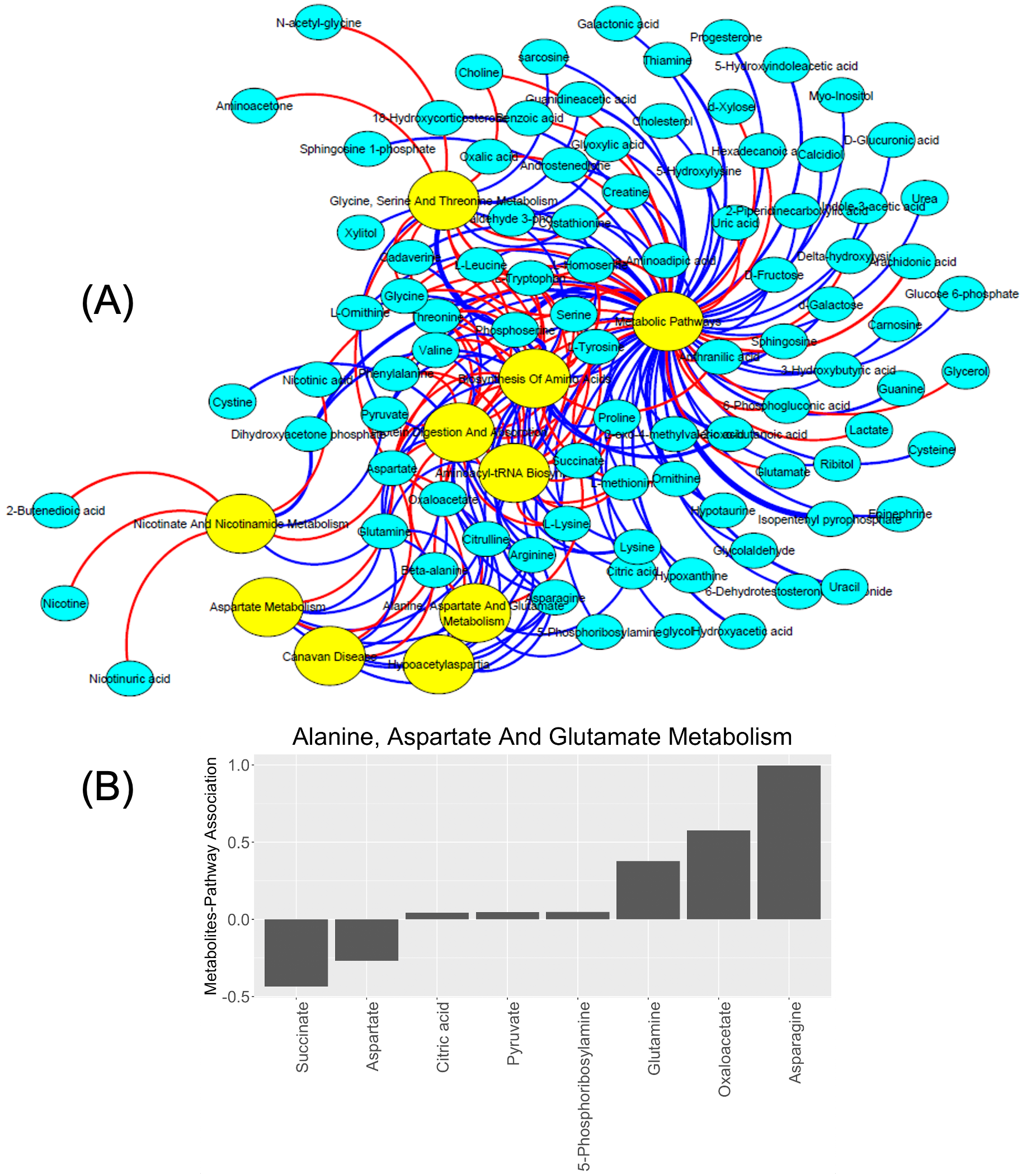

Supplement: giaa162_Supplemental_Files [file giaa162_supplemental_files.zip › Lilikoi Figure 5.docx]
