# Supplementary material for: Lilikoi V2.0: a deep learning–enabled, personalized pathway-based R package for diagnosis and prognosis predictions using metabolomics data
Source: Gigascience. 2021 Jan 23;10(1):giaa162. doi: 10.1093/gigascience/giaa162 (PMC7825009; doi:10.1093/gigascience/giaa162)
Supplement: giaa162_Response_to_Reviewer_Comments_Original_Submission [file giaa162_response_to_reviewer_comments_original_submission.pdf]

Author's Response To Reviewer Comments

Close

Answers to Reviewers:

Reviewer #1: The manuscript with ID GIGA-D-20-00214, entitled "Lilikoi V2.0: a deep-learning enabled, personalized pathway-based R package for diagnosis and prognosis predictions using metabolomics data" submitted by Xinying Fang; Yu Liu; Zhijie Ren; Yuheng Du; Qianhui Huang; Lana Garmire et al. to the journal GigaScience introduces the version 2 of their DL -enabled metabolomic pathway analysis package Lilikoi. In deed innovative and interesting approach. Indeed a very important and timely topic. However, the following comments / suggestions/ advice are provided to help the authors improve on the currently submitted version of the manuscript only.

Critique and scope to improve
A table showing the comparison against currently popular pathway analysis tools and Lilikoi V1, for metabolomics-scale data, their in put output, ease of use, time of analysis, strengths and short comings, visualization types, statistical rigor, pre-processing capabilities, dependencies, would aid the reader to capture the current state of the art in the field.

Thank you for the suggestion. As far as we know, Lilikoi is the first package that offers personalized (or individualized) pathway scores, making it very distinct from other popular metabolomics enrichment analysis tools. Another earlier paper by Marco-Ramell etc [1] had done comprehensive comparisons on 13 methods, which were different variations of Fisher's exact tests or hypergeometric tests. We therefore refer the readers to this benchmark study to avoid repetitions.
Refrenece: [1]. Marco-Ramell A, Palau-Rodriguez M, Alay A, Tulipani S, Urpi-Sarda M, Sanchez-Pla A, et al. Evaluation and comparison of bioinformatic tools for the enrichment analysis of metabolomics data. BMC Bioinformatics [Internet]. BioMed Central; 2018 [cited 2020 Nov 17];19. Available from: https://www.ncbi.nlm.nih.gov/pmc/articles/PMC5749025/

This reviewer faced issues installing lilikoi upfront:
"Warning message:
package 'lilikoi' is not available (for R version 4.0.2)"
and also
"Warning message:
package 'lilikoi' is not available (for R version 3.5.2)"
These may be resolved by the authors too.
The dependencies on Python, R and other packages are not clearly mentioned/ listed in the MS.
Our apologies. The link to Lilikoi was not approved by CRAN team at the time of last review, but it is updated and available on CRAN now: https://cran.r-project.org/web/packages/lilikoi/index.html. We also mentioned clearly the dependencies in the manuscript revision (Method section).

The tool will greatly benefit with PDF stepwise and detailed manual which is clearly missing.
Thank you for the suggestion. We attach the manual as the supplementary material. It is also available in CRAN R package.

Even using the Shiny tool (https://lilikoi.shinyapps.io/lilikoi/) specified "sample data" leads to error messages such as "An error has occurred. Check your logs or contact the app author for clarification." Under : "feature mapping" tab. Plus, the next steps on Shinny are extremely slow, and are time-intensive in this reviewers experience. A lot of scope to improve.
Our apologies. The Shiny app was for the older version. Users are now strongly encouraged to use/download Lilikoi v2 as the R package and on the new Github website for Lilikoi v2.

Too elaborate conclusion, keep it crisp and clear with a strong message. Unsure how NLP will improve any of these.
Thank you for the suggestion. We have now removed NLP mentioning in the revision.

Authors should also come up with recommendations that would guide the users of their tool, for instance:
-which of the DL, GBM, LDA, LOG, RF, RPART, SVM perform optimally for LC-MS vs GC-MS vs NMR data?
Thank you for the good question. It is very difficult, if ever possible, to make a generalized recommendation, as the results are really data and sample size dependent. As a rule of thumb, we do recommend users in the revision, to pay attentions to machine learning methods that are less prone to overfitting (such as random forest, or RF)

-Which options are "Default" before reaching to the prognosis model from the input data?
Thank you for the questions. We have clarified these in the revision, whenever default is needed.
For Cox-nnet, the default optimization method is the standard gradient descent.
For Cox-PH, the default lambda parameter for prediction in Cox-PH is lambda.1se. The default penalization method alpha is 1, which is the Lasso penalization.

Describe what kind of computational resources one user needs to perform all these analysis, and how many datasets can Lilikoi v2 handle and in what time? Any bench marking done against any other comparable tools?
Version of R: 3.5.1 The users can run the job on the desktop or on the server. Deep learning is more time-consuming on the desktop with specs of macOS with a processor of 2.6 GHz 6-Core Intel Core i7 and memory of 16 GB 2667 MHz DDR4. It takes about 15 mins for each run.
Additionally, we have listed the running time for each machine-learning method as an additional field in Table 1.
The closest comprehensive packages that we know is MetaboAnalystR. Some functions are similar between the two, such as classification using caret packages, however, there are some very significant differences between the two as well, making it impossible to benchmarking, For example, deep-learning modules are unique and only available to our package, to our knowledge. We also have modules for prognosis prediction, which is unique. The individualized pathway calculation is our new offering. On the other hand, MetaboAnalystR provides other functionalities, such as time-series analysis, power analysis, and network explorer. We added the comparisons between them in the discussion section.

Terms such as "pathway deregulation" and " "pathway dysregulation" need to be defined, contextualized for the readers.
Apologies. The correct term should be "pathway dysregulation". We corrected in the revision, and added the definition: “PDS is a normalized score between [0,1] that measures the degree of dysregulation of a pathway relative to the norm (controls)”.

Table 1, for some metrics why does the "testing" has a better score than the "training" data set?
Thank you for the comment. The very small differences (0.0x) between testing and training data are presumably due to the randomness in the seeds that were used to split the training and testing data. We did statistical tests among the runs, and found that none of the differences in the metrics are significant.

The authors have smartly used openly available metabolomics datasets for the tool development/validation which is appreciable.In the "Datasets" section specify as to which platforms, LC-MS, GC-MS were used to generate the original datasets besides the accession numbers will help readers relating with the platform capabilities and data structure/size.
Thank you for your encouragement. We now have revised the Datasets section of the manuscript to added the platforms and the number of metabolites, whenever they were reported by the original publications (in the first 2 datasets).

Unsure why accessing the CRAN package this reviewer gets these messages:
"Package 'lilikoi' was removed from the CRAN repository.
Formerly available versions can be obtained from the archive.
Archived on 2020-07-22 for policy violation.
Please use the canonical form https://CRAN.R-project.org/package=lilikoi to link to this page."
Sorry, this problem is now fixed and the package is available (at the time of this revision submission).
We had some issue with lilikoi v1 maintenance due to the departure of a previous group member, we have now designated the PI as the contacting person responsible for the active maintenance of the package.

Table 1. Is this is the sole "performance" defining analysis? What's the time-taken for analysis for each of these on the 3 datasets used?

Thank you for the good question! We have added the running time as additional performance metric in Table 1.

Figure 1 lacks details: Should specify what "input data" format, what does "Feature mapper" do?, which Normalization methods to choose, which imputation methods apply. Without granular informations it hard to find usefulness of the workflow.
Thank you for the comment. We have added more explanation in the figure 1, particularly, we added: “Input data requires metabolomics data matrix, and one column of a categorical variable to specify case/control status for each subject (for classification), or survival information (for prognosis analysis). Feature mapping converts metabolite names to standardized metabolic IDs (e.g. HMDB IDs) and then transforms them into pathway names. Pre-processing enables three normalization methods (standard normalization, quantile normalization and median-fold normalization) and one knn imputation method.”

Also, Figure 1 indicates that "classification and "prognosis models" do not interact?
Yes, They are treated as two different types of models: classification models vs. survival models, since the meta data are different. Classification needs class labels, and survival model needs time-to-event survival information.

Figure 2A, B are not well explained in the text in terms of their significance and statistically important metrices.
Thank you for your suggestions. We have explained the metrics better in the figure legend: “AUC, or accuracy, measures how the model can distinguish between classes. SEN, or Sensitivity, measures the capability of a model to correctly identify cases or diseases. SPEC, or Specificity, measures the capability of a model to correctly identify controls or normal status. F1-statistics measures the accuracy of a model. Balanced accuracy is the mean of specificity and sensitivity, a good metrics to consider when the sample sizes in cases and controls are not balanced.”

Figure 4 might be representative but the data is too sparse in terms of the # of metabolites mapped?
Yes, this is a general issue of metabolomics technology, we usually see 200-300 metabolites in a data set, and there are some missing metabolites (about 20%) in the pathways.

Figure 5 needs to improve, unreadable, too many over lapping nodes, can not read the metabolites/ pathways or have been cut out. Too many characters " ' " ' in those names that need a clean up. Legend with colour and node size, edge descriptions are missing too. Also, in the inset with the small bar graph, what's the Y-axis label ?
Thank you for the suggestions. We removed the characters “`”. We modified the layout and the size for the nodes, and made metabolites/pathway names more clear..

The supplementary document "er_162" should provide the various metabolite Identifiers such as KEGG, Metlin, HMDB, SMILES, InChiKeys for future facilitation of studies on this dataset. Provide the list of qualifier/ quantifier ions, retention times (RTs) MS/MS spectral matrix (intensity and m/z) for aiding the readers.
Same comment as above for "JCI71180sd2_processed_tumor" and "plasma_breast_cancer" and "JCI71180sd2_processed_lilikoi"documents.
Thank you for the suggestions. We have added different IDs for metabolites in the new csv files. The “JCI71180sd2_processed_tumor” file is the tumor sample subset of “JCI71180sd2_processed_lilikoi” file, so we just uploaded the identifier file for the “JCI71180sd2_processed_lilikoi” file. The original datasets didn’t have M/Z score, so we couldn’t add them.

Moderate linguistic (English) corrections are desirable. Present version has some common, punctuation, tense, grammatical, typological sentence framing/ phrase construction issues which should be taken care by the authors.
Thanks. We did do additional rounds of proofreading to improve them.

Please change and improve upon the conclusion to provide very specific and key take-home messages to the readers and should be only based on the findings and not extrapolations/ future aspects.
Thank you. We have improve the conclusion section with additional take-home messages from the results. We removed extrapolations and future aspects.

References are NOT OK and not well organized in terms of formats. Follow the journal format typically as instructed to the authors. Please be consistent.Check all the references one by one manually, so that

everything cited in the text are also listed in the list of references.

Thank you. We have done reference checking more carefully.

If at all a revised version is submitted, then please make all changes in differently colored fonts (red/blue) or highlighted background (yellow) so that it would save the reviewer's time in finding the changes with lesser efforts and time.
We have used blue font in the revision, for the changes we made.

Please also incorporate all the answers/ discussions/ points raised by this reviewer into the manuscript rather than just explaining to this reviewer- in order to make the manuscript stronger than it is now.

We are including the point-to-point answers to reviewers, along with the revision.

Reviewer #2: The studies reported in this manuscript provide an updated version of Lilikoi. This new version software includes several new features, including deep-learning classification, prognosis prediction,
data preprocessing, and metabolite-pathway regression. A large amount of data was used for model training and testing. Compared with its previous version, this new version is capable of more comprehensive analysis. This manuscript has been well prepared. Some minor revisions are needed to improve its quality.

(1) Data from different types of samples, such as plasma and tissue, were used to train and test models, but it is unclear how they were correlated or integrated. Apparently, metabolites from plasma and tissues have different compositions and abundances.
Sorry for the confusion. The three data sets were used to address objectives in 3 different sections in the results. In each result section, the train and test dataset are subsetted from the same dataset.

(2) Prognosis prediction is an important new feature; however, it is unclear how metabolomics data and prognosis prediction methods were integrated. It seems that "The input parameters are event (eg. death), survival time and penalized covariates" is for the prediction methods, whereas the input from metabolites was not clearly described.
Thank you for the question. The input data are metabolite data as well as the time to event data. We have clarified this in revision (Fig 1 legend).

(3) Language issues. The following sentences need to be revised. "For imputation, we used k-nearest neighbors method…", in which the abbreviation "knn" is missing. "F-1 statistic is a good unbiased metric given the unbalanced samples in ER= and ER- classes" has a typo "=". "The reduction of aspartate in ER- patients is consistent with observation before."; it should be "previous observation". "metabolites significantly ($p<0.05$) associated with the pathways. show how each metabolite contributes to". "The generic term "metabolic pathways", is associated with the largest number of metabolites." In Conclusion, "Such endeavor sets Lilikoi apart from other more conventional metabolomics analysis packages"; it seemed that authors meant Lilikoi 2.0.
Thanks for the comments. We have done additional rounds of revision. We fixed the typos as the reviewer pointed out.

Close
