# Supplementary material for: Lilikoi V2.0: a deep learning–enabled, personalized pathway-based R package for diagnosis and prognosis predictions using metabolomics data
Source: Gigascience. 2021 Jan 23;10(1):giaa162. doi: 10.1093/gigascience/giaa162 (PMC7825009; doi:10.1093/gigascience/giaa162)
Supplement: giaa162_Response_to_Reviewer_Comments_Revision_1 [file giaa162_response_to_reviewer_comments_revision_1.pdf]

Author's Response To Reviewer Comments

Close

Dear Editor,
Thank you for the guideline on sending the marked up word.docx version of our latest manuscript with your comments. We have filled in the several sections that were previously missing, such as Funding, Competing interests etc.
Please let me know if you need anything else from me.
Best
Lana Garmire

Close
