# Supplementary material for: Lilikoi V2.0: a deep learning–enabled, personalized pathway-based R package for diagnosis and prognosis predictions using metabolomics data
Source: Gigascience. 2021 Jan 23;10(1):giaa162. doi: 10.1093/gigascience/giaa162 (PMC7825009; doi:10.1093/gigascience/giaa162)
Supplement: giaa162_Reviewer_1_Report_Original_Submission — Biswapriya Misra -- 8/15/2020 Reviewed [file giaa162_reviewer_1_report_original_submission.pdf]

Reviewer Report

Title: Lilikoi V2.0: a deep-learning enabled, personalized pathway-based R package for diagnosis and prognosis predictions using metabolomics data

Version: Original Submission **Date:** 8/15/2020

Reviewer name: Biswapriya Misra

Reviewer Comments to Author:

The manuscript with ID GIGA-D-20-00214, entitled "Lilikoi V2.0: a deep-learning enabled, personalized pathway-based R package for diagnosis and prognosis predictions using metabolomics data" submitted by Xinying Fang; Yu Liu; Zhijie Ren; Yuheng Du; Qianhui Huang; Lana Garmire et al. to the journal GigaScience introduces the version 2 of their DL -enabled metabolomic pathway analysis package Lilikoi. In deed innovative and interesting approach. Indeed a very important and timely topic. However, the following comments / suggestions/ advice are provided to help the authors improve on the currently submitted version of the manuscript only.
Critique and scope to improve
A table showing the comparison against currently popular pathway analysis tools and Lilikoi V1, for metabolomics-scale data, their in put output, ease of use, time of analysis, strengths and short comings, visualization types, statistical rigor, pre-processing capabilities, dependencies, would aid the reader to capture the current state of the art in the field.
This reviewer faced issues installing lilikoi upfront:
"Warning message:
package 'lilikoi' is not available (for R version 4.0.2)"
and also
"Warning message:
package 'lilikoi' is not available (for R version 3.5.2)"
These may be resolved by the authors too.
The dependencies son Python, R and other packages are not clearly mentioned/ listed in the MS.
The tool will greatly benefit with PDF stepwise and detailed manual which is clearly missing.
Even using the Shiny tool (https://lilikoi.shinyapps.io/lilikoi/) specified "sample data" leads to error messages such as "An error has occurred. Check your logs or contact the app author for clarification."
Under : "feature mapping" tab.
Plus, the next steps on Shinny are extremely slow, and are time-intensive in this reviewers experience. A lot of scope to improve.
Too elaborate conclusion, keep it crisp and clear with a strong message. Unsure how NLP will improve any of these.
Authors should also come up with recommendations that would guide the users of their tool, for instance:
-which of the DL, GBM, LDA, LOG, RF, RPART, SVM perform optimally for LC-MS vs GC-MS vs NMR data?
-Which options are "Default" before reaching to the prognosis model from the input data?

Describe what kind of computational resources one user needs to perform all these analysis, and how many datasets can Lilikoi v2 handle and in what time? Any bench marking done against any other comparable tools ?
Terms such as "pathway deregulation" and " "pathway dysregulation" need to be defined, contextualized for the readers.
Table 1, for some metrics why does the "testing" has a better score than the "training" data set?
The authors have smartly used openly available metabolomics datasets for the tool development/ validation which is appreciable.
In the "Datasets" section specify as to which platforms, LC-MS, GC-MS were used to generate the original datasets besides the accession numbers will help readers relating with the platform capabilities and data structure/size.
Unsure why accessing the CRAN package this reviewer gets these messages:
"Package 'lilikoi' was removed from the CRAN repository.
Formerly available versions can be obtained from the archive.
Archived on 2020-07-22 for policy violation.
Please use the canonical form https://CRAN.R-project.org/package=lilikoi to link to this page."
Table 1. Is this is the sole "performance" defining analysis? What's the time-taken for analysis for each of these on the 3 datasets used?
Figure 1 lacks details: Should specify what "input data" format, what does "Feature mapper" do?, which Normalization methods to choose, which imputation methods apply. Without granular informations it hard to find usefulness of the workflow.
Also, Figure 1 indicates that "classification and "prognosis models" do not interact?
Figure 2A, B are not well explained in the text in terms of their significance and statistically important metrices.
Figure 4 might be representative but the data is too sparse in terms of the # of metabolites mapped?
Figure 5 needs to improve, unreadable, too many over lapping nodes, can not read the metabolites/ pathways or have been cut out. Too many characters " ' " ' in those names that need a clean up. Legend with colour and node size, edge descriptions are missing too. Also, in the inset with the small bar graph, what's the Y-axis label ?
The supplementary document "er_162" should provide the various metabolite Identifiers such as KEGG, Metlin, HMDB, SMILES, InChiKeys for future facilitation of studies on this dataset. Provide the list of qualifier/ quantifier ions, retention times (RTs) MS/MS spectral matrix (intensity and m/z) for aiding the readers.
Same comment as above for "JCI71180sd2_processed_tumor" and "plasma_breast_cancer" and "JCI71180sd2_processed_lilikoi"documents.
Moderate linguistic (English) corrections are desirable. Present version has some common, punctuation, tense, grammatical, typological sentence framing/ phrase construction issues which should be taken care by the authors.
Please change and improve upon the conclusion to provide very specific and key take-home messages to the readers and should be only based on the findings and not extrapolations/ future aspects.
References are NOT OK and not well organized in terms of formats. Follow the journal format typically as instructed to the authors. Please be consistent.Check all the references one by one manually, so that

everything cited in the text are also listed in the list of references.
If at all a revised version is submitted, then please make all changes in differently colored fonts (red/blue) or highlighted background (yellow) so that it would save the reviewer's time in finding the changes with lesser efforts and time.
Please also incorporate all the answers/ discussions/ points raised by this reviewer into the manuscript rather than just explaining to this reviewer- in order to make the manuscript stronger than it is now.

Methods

Are the methods appropriate to the aims of the study, are they well described, and are necessary controls included? Choose an item.

Conclusions

Are the conclusions adequately supported by the data shown? Choose an item.

Reporting Standards

Does the manuscript adhere to the journal's guidelines on minimum standards of reporting? Choose an item.

Choose an item.

Statistics

Are you able to assess all statistics in the manuscript, including the appropriateness of statistical tests used? Choose an item.

Quality of Written English

Please indicate the quality of language in the manuscript: Choose an item.

Declaration of Competing Interests

Please complete a declaration of competing interests, considering the following questions:

- Have you in the past five years received reimbursements, fees, funding, or salary from an organisation that may in any way gain or lose financially from the publication of this manuscript, either now or in the future?
- Do you hold any stocks or shares in an organisation that may in any way gain or lose financially from the publication of this manuscript, either now or in the future?
- Do you hold or are you currently applying for any patents relating to the content of the manuscript?

- Have you received reimbursements, fees, funding, or salary from an organization that holds or has applied for patents relating to the content of the manuscript?
- Do you have any other financial competing interests?
- Do you have any non-financial competing interests in relation to this paper?

If you can answer no to all of the above, write 'I declare that I have no competing interests' below. If your reply is yes to any, please give details below.

I declare that I have no competing interests.

I agree to the open peer review policy of the journal. I understand that my name will be included on my report to the authors and, if the manuscript is accepted for publication, my named report including any attachments I upload will be posted on the website along with the authors' responses. I agree for my report to be made available under an Open Access Creative Commons CC-BY license (http://creativecommons.org/licenses/by/4.0/). I understand that any comments which I do not wish to be included in my named report can be included as confidential comments to the editors, which will not be published.

Choose an item.

To further support our reviewers, we have joined with Publons, where you can gain additional credit to further highlight your hard work (see: https://publons.com/journal/530/gigascience). On publication of this paper, your review will be automatically added to Publons, you can then choose whether or not to claim your Publons credit. I understand this statement.

Yes Choose an item.
