# Supplementary material for: Lilikoi V2.0: a deep learning–enabled, personalized pathway-based R package for diagnosis and prognosis predictions using metabolomics data
Source: Gigascience. 2021 Jan 23;10(1):giaa162. doi: 10.1093/gigascience/giaa162 (PMC7825009; doi:10.1093/gigascience/giaa162)
Supplement: giaa162_Reviewer_2_Report_Original_Submission — Zhibo Yang -- 8/23/2020 Reviewed [file giaa162_reviewer_2_report_original_submission.pdf]

Reviewer Report

Title: Lilikoi V2.0: a deep-learning enabled, personalized pathway-based R package for diagnosis and prognosis predictions using metabolomics data

Version: Original Submission **Date:** 8/23/2020

Reviewer name: Zhibo Yang

Reviewer Comments to Author:

The studies reported in this manuscript provide an updated version of Lilikoi. This new version software includes several new features, including deep-learning classification, prognosis prediction, data preprocessing, and metabolite-pathway regression. A large amount of data was used for model training and testing. Compared with its previous version, this new version is capable of more comprehensive analysis. This manuscript has been well prepared. Some minor revisions are needed to improve its quality.

(1) Data from different types of samples, such as plasma and tissue, were used to train and test models, but it is unclear how they were correlated or integrated. Apparently, metabolites from plasma and tissues have different compositions and abundances.

(2) Prognosis prediction is an important new feature; however, it is unclear how metabolomics data and prognosis prediction methods were integrated. It seems that "The input parameters are event (eg. death), survival time and penalized covariates" is for the prediction methods, whereas the input from metabolites was not clearly described.

(3) Language issues. The following sentences need to be revised. "For imputation, we used k-nearest neighbors method…", in which the abbreviation "knn" is missing. "F-1 statistic is a good unbiased metric given the unbalanced samples in ER= and ER- classes" has a typo "=". "The reduction of aspartate in ER- patients is consistent with observation before."; it should be "previous observation". "metabolites significantly (p<0.05) associated with the pathways. show how each metabolite contributes to". "The generic term "metabolic pathways", is associated with the largest number of metabolites." In Conclusion, "Such endeavor sets Lilikoi apart from other more conventional metabolomics analysis packages"; it seemed that authors meant Lilikoi 2.0.

Methods

Are the methods appropriate to the aims of the study, are they well described, and are necessary controls included? Choose an item.

Conclusions

Are the conclusions adequately supported by the data shown? Choose an item.

Reporting Standards

Does the manuscript adhere to the journal's guidelines on minimum standards of reporting? Choose an item.

Choose an item.

Statistics

Are you able to assess all statistics in the manuscript, including the appropriateness of statistical tests used? Choose an item.

Quality of Written English

Please indicate the quality of language in the manuscript: Choose an item.

Declaration of Competing Interests

Please complete a declaration of competing interests, considering the following questions:

- Have you in the past five years received reimbursements, fees, funding, or salary from an organisation that may in any way gain or lose financially from the publication of this manuscript, either now or in the future?
- Do you hold any stocks or shares in an organisation that may in any way gain or lose financially from the publication of this manuscript, either now or in the future?
- Do you hold or are you currently applying for any patents relating to the content of the manuscript?
- Have you received reimbursements, fees, funding, or salary from an organization that holds or has applied for patents relating to the content of the manuscript?
- Do you have any other financial competing interests?
- Do you have any non-financial competing interests in relation to this paper?

If you can answer no to all of the above, write 'I declare that I have no competing interests' below. If your reply is yes to any, please give details below.

I declare that I have no competing interests.

I agree to the open peer review policy of the journal. I understand that my name will be included on my report to the authors and, if the manuscript is accepted for publication, my named report including any attachments I upload will be posted on the website along with the authors' responses. I agree for my report to be made available under an Open Access Creative Commons CC-BY license (http://creativecommons.org/licenses/by/4.0/). I understand that any comments which I do not wish to be included in my named report can be included as confidential comments to the editors, which will not be published.

Choose an item.

To further support our reviewers, we have joined with Publons, where you can gain additional credit to further highlight your hard work (see: https://publons.com/journal/530/gigascience). On publication of this paper, your review will be automatically added to Publons, you can then choose whether or not to claim your Publons credit. I understand this statement.

Yes Choose an item.
